# Ecological Factors Generally Not Altitude Related Played Main Roles in Driving Potential Adaptive Evolution at Elevational Range Margin Populations of Taiwan Incense Cedar (*Calocedrus formosana*)

**DOI:** 10.3389/fgene.2020.580630

**Published:** 2020-11-11

**Authors:** Wei-Ming Chien, Chung-Te Chang, Yu-Chung Chiang, Shih-Ying Hwang

**Affiliations:** ^1^School of Life Science, National Taiwan Normal University, Taipei, Taiwan; ^2^Department of Life Science, Tunghai University, Taichung, Taiwan; ^3^Department of Biological Sciences, National Sun Yat-sen University, Kaohsiung, Taiwan

**Keywords:** adaptive evolution, AFLP, allele frequency, *Calocedrus formosana*, elevational range margin populations, environment, gene flow

## Abstract

Population diversification can be shaped by a combination of environmental factors as well as geographic isolation interacting with gene flow. We surveyed genetic variation of 243 samples from 12 populations of *Calocedrus formosana* using amplified fragment length polymorphism (AFLP) and scored a total of 437 AFLP fragments using 11 selective amplification primer pairs. The AFLP variation was used to assess the role of gene flow on the pattern of genetic diversity and to test environments in driving population adaptive evolution. This study found the relatively lower level of genetic diversity and the higher level of population differentiation in *C. formosana* compared with those estimated in previous studies of conifers including *Cunninghamia konishii*, *Keteleeria davidiana* var. *formosana*, and *Taiwania cryptomerioides* occurring in Taiwan. BAYESCAN detected 26 *F*_ST_ outlier loci that were found to be associated strongly with various environmental variables using multiple univariate logistic regression, latent factor mixed model, and Bayesian logistic regression. We found several environmentally dependent adaptive loci with high frequencies in low- or high-elevation populations, suggesting their involvement in local adaptation. Ecological factors, including relative humidity and sunshine hours, that are generally not altitude related could have been the most important selective drivers for population divergent evolution in *C. formosana*. The present study provides fundamental information in relation to adaptive evolution and can be useful for assisted migration program of *C. formosana* in the future conservation of this species.

## Introduction

Linking ecology with evolutionary biology is important to understand how environments drive adaptive divergence among populations within a species’ distribution range. Gene dispersal is a vital process that maintains genetic diversity and is critical to population resilience and persistence (Savolainen et al., 2007; Kremer et al., 2012) because genetic variation plays a key role in a population that is robust to future environmental changes (Reed and Frankham, 2003). Environmental heterogeneity apart from geographic isolation can impose strong constraints on gene flow among populations of a species and locally adapted fitness-related traits can evolve driven by selection (Kawecki and Ebert, 2004; Alberto et al., 2013). Migration of pre-adapted alleles can play roles in local adaptation of sink populations (Hamrick, 2004; Kremer et al., 2012). However, gene flow among populations inhabiting environmentally distinct areas can have swamping effects due to the disruption of locally adaptive gene complex or the incursion of maladapted alleles (Kirkpatrick and Barton, 1997; Lenormand, 2002; Sexton et al., 2014).

If a species’ distribution covers only a small elevational band in a small latitudinal range, population adaptive divergence can be precluded due to the effect of gene flow and divergence between populations may be mainly result from drift-mediated process (García-Ramos and Kirkpatrick, 1997; Conner and Hartl, 2004; Li et al., 2018). However, population environmental conditions of a species can vary dramatically within a small latitudinal range (Fang et al., 2013; Huang et al., 2015a,b; Shih et al., 2018; Li et al., 2019). Patterns of genetic diversity can exist in a species along elevational gradients of its distribution (Ohsawa and Ide, 2008; Halbritter et al., 2015). Theory predicts that diversification and adaptation may be accelerated at the elevational edges of a species’ distribution range (Funk et al., 2005; Hodkinson, 2005; Halbritter et al., 2015). Identification of high frequency eco-alleles associated with environmental factors can be crucial in the assisted migration program for species conservation, particularly in the face of global climate change (Aitken et al., 2008; Aitken and Whitlock, 2013; Aitken and Bemmels, 2016).

Environmentally dependent adaptive fingerprints have been widely detected in conifers (e.g., Mimura and Aitken, 2010; Grivet et al., 2011; Chen et al., 2012; Fang et al., 2013; Shih et al., 2018; Li et al., 2019). Contrasting patterns of adaptive divergence or neutral evolution were observed in coniferous species occurring in Taiwan such as *Keteleeria davidiana* var. *formosana* (Fang et al., 2013), *Taiwania cryptomerioides* (Li et al., 2018), and *Cunninghamia konishii* (Li et al., 2019). No population adaptive divergence was detected among Taiwanese populations of *T. cryptomerioides* (Li et al., 2018). In contrast, population adaptive divergence associated with environmental conditions was found in *K. davidiana* var. *formosana* and *C. konishii* (Fang et al., 2013; Shih et al., 2018; Li et al., 2019). These contrasting evolutionary patterns might have been related to the patterns of distribution of these species encompassing different geographic areas and elevational ranges. *T. cryptomerioides* in Taiwan is distributed in a smaller elevational range (1800–2400 m, Li and Keng, 1994b; Li et al., 2018) compared to *C. konishii*, which is distributed in a wider elevational band (1000–2500 m, Liu, 1966; Li et al., 2019). *K. davidiana* var. *formosana*, though occupying only a small elevational range (300–800 m), is distributed in geographically distant areas restricted in northern and southern Taiwan (Li and Keng, 1994c).

Genetic variation can be quantified using amplified fragment length polymorphism (AFLP), representing DNA sequence variation, for non-model organisms. AFLP can be used in testing patterns of genetic differentiation influenced by gene flow and environments (Chen et al., 2012; Fang et al., 2013; Huang et al., 2015a,b; Li et al., 2018, 2019). Other genotyping methods such as expressed sequence tags, single nucleotide polymorphisms can also be used in investigating the relationship between genetic variation and environments (e.g., Hsieh et al., 2013; Huang et al., 2016; Shih et al., 2018). We used AFLP in this study because it can be easily applied in non-model organisms and hundreds of potential loci can be genotyped across the genome (Paun and Schönswetter, 2012). Gene flow among populations of a species’ distribution range can be explained by the models of isolation by distance (IBD) (Wright, 1943) and isolation by environment (IBE) (Wang et al., 2013; Sexton et al., 2014). Geographical distance can be the primary constraint on gene dispersal, and a pattern of IBD arise. Alternatively, IBE predicts that habitats over environmental gradients where natural selection rather than neutral drift is the evolutionary driving force for divergence. Disentangling the effects of IBE and IBD acting on the divergence of conspecific populations is critical to understanding the process of how environments shape population divergence (Shafer and Wolf, 2013; Sexton et al., 2014; Huang et al., 2016) and for species conservation as biodiversity increasingly threatened by global change (Hoffmann and Sgrò, 2011; Alberto et al., 2013; D’Amen et al., 2013; Lavergne et al., 2013).

**FIGURE 1:**
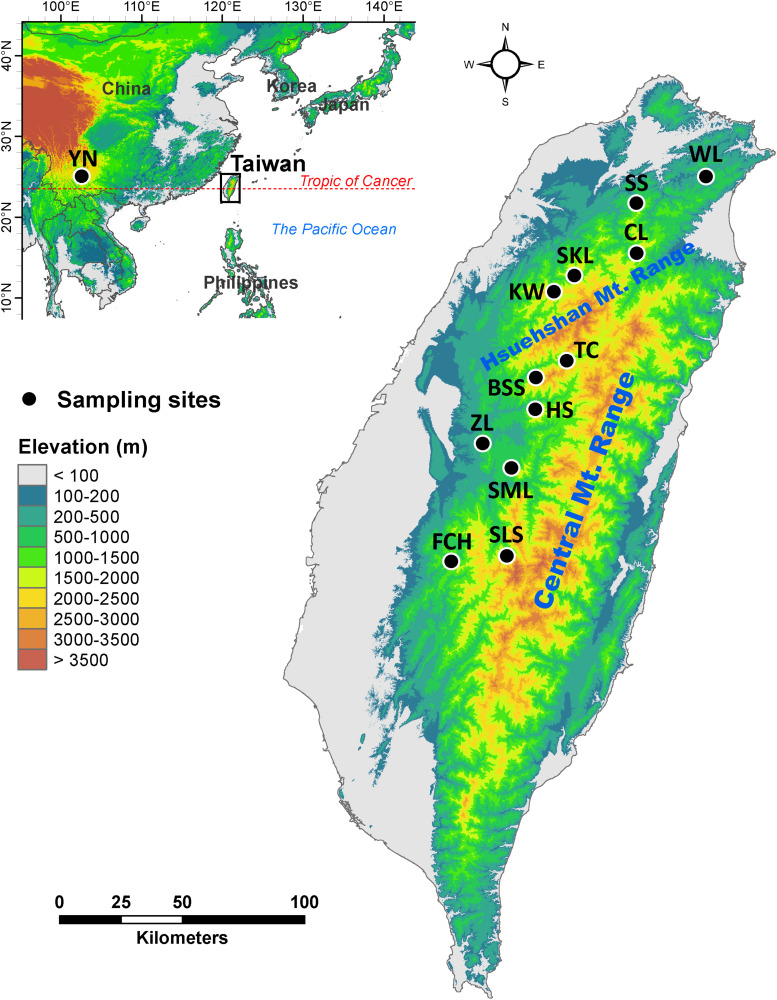
Geographic distribution of the 12 populations of *Calocedrus formosana* occur in Taiwan. See Table 1 for abbreviations of the 12 populations of *C. formosana*.

Mountainous regions in Taiwan constituting forest vegetation zones based on local climate primarily driven by altitude (Su, 1984; Li et al., 2013), ranging from deep valleys to high mountain peaks, with rugged topography and steep elevational environmental gradients (Su, 1984; Li et al., 2013), are biodiversity hotspots and important reservoirs of genetic diversity. *Calocedrus formosana* is distributed in a wide geographic area along an elevational band from 300 to 2200 m (Li and Keng, 1994a; Figure 1 and Table 1). The most important feature of *C. formosana*, compared to other conifers occur in Taiwan is its wider elevational distribution, and this makes it an exemplar coniferous species for the study of adaptive evolution along an elevational gradient. *C. formosana* populations distributed altitudinally in three different forest vegetation zones: sub-montane evergreen zone (SME, 0–800 m), montane evergreen cloud zone (ME, 800–1400 m), and montane mixed cloud zone (MMC, 1400 m∼) (Su, 1984; Li et al., 2013). Mountains can act as barriers to gene flow influencing patterns of biogeographic differentiation of a species (Antonelli, 2017) and were found to have had an active role in restricting gene flow in *C. konishii* (Li et al., 2019). Geographically, populations of *C. formosana* can be separated into areas north and south of the Hsuehshan mountain range (HMR, Figure 1). The HMR may act as a barrier hindering gene flow between populations of *C. formosana* north and south of this mountain range. In addition, environments in different forest vegetation zones might have also played roles in shaping population differentiation.

**TABLE 1:** Site properties and genetic parameters of the sampled *Calocedrus formosana* populations.

**Locality**	**Latitude/Longitude**	**Altitude (m)**	***N***	**Forest vegetation zone**	***F*_IS_ (95% CI)**	**%*P***	***uH*_E_ (SE)**	***I*_A_ (*P*)**	***r*D (*P*)**
Bashienshan (BSS)	24.18555/121.0386	1352	28	MEC	0.509 (0.026, 0.977)	40.5	0.138 (0.008)	1.919 (0.001)	0.0101 (0.001)
Chilan (CL)	24.64227/121.4458	1136	10	MEC	0.497 (0.029, 0.976)	50.8	0.172 (0.009)	3.530 (0.001)	0.0198 (0.001)
Fengchihu (FCH)	23.50638/120.7006	1518	23	MMC	0.508 (0.024, 0.977)	36.6	0.104 (0.007)	1.586 (0.001)	0.0117 (0.001)
Huisun (HS)	24.06833/121.0361	1243	17	MEC	0.497 (0.026, 0.974)	39.6	0.135 (0.008)	2.302 (0.001)	0.0143 (0.001)
Kuanwu (KW)	24.50111/121.1103	2155	14	MMC	0.506 (0.029, 0.977)	43.7	0.175 (0.009)	5.836 (0.001)	0.0262 (0.001)
Siakelo (SKL)	24.56111/121.1936	2209	30	MMC	0.506 (0026, 0.976)	45.8	0.159 (0.009)	2.897 (0.001)	0.0129 (0.001)
Salishien (SLS)	23.52777/120.9208	1220	12	MEC	0.505 (0.026, 0.975)	42.6	0.159 (0.009)	1.295 (0.002)	0.0077 (0.002)
Sunmoonlake (SML)	23.85138/120.9394	859	20	MEC	0.505 (0027, 0.978)	39.4	0.125 (0.008)	1.107 (0.001)	0.0072 (0.001)
Sansia (SS)	24.82750/121.4456	436	7	SME	0.505 (0.028, 0.975)	43.5	0.153 (0.009)	0.423 (0.124)	0.0035 (0.124)
Techi (TC)	24.24666/121.1622	1498	35	MMC	0.499 (0.026, 0.971)	42.3	0.154 (0.009)	17.779 (0.001)	0.0710 (0.001)
Wulai (WL)	24.92444/121.7267	562	15	SME	0.498 (0.025, 0.975)	41.0	0.145 (0.009)	0.797 (0.001)	0.0051 (0.001)
Zhingliao (ZL)	23.94138/120.8244	868	32	MEC	0.498 (0.024, 0.979)	39.1	0.135 (0.008)	3.641 (0.001)	0.0190 (0.001)
Total			243						
Average			20.3			42.1	0.146 (0.006)		

*N, Number of samples; F_*IS*_, inbreeding coefficient and confidence interval (95% CI) estimated using the f-free model of the HICKORY program (Holsinger and Lewis, 2003); SME, sub-montane evergreen zone (0-800 m); MEC, montane evergreen cloud zone (800-1400 m); MMC, montane mixed cloud zone (1400 m∼); %P, the percentage of polymorphic loci; uH_*E*,_ unbiased expected heterozygosity. I_*A*_, index of association; rD, modified index of association.*

High elevation populations are typically more vulnerable to environmental change than their low elevation counterparts because of range restriction and dispersal limitations (Savolainen et al., 2007; Allendorf et al., 2010; Hoffmann and Sgrò, 2011). In addition, it was pointed out by Körner (2007) that environmental variables can be classified into two categories: those that are altitude-related, such as temperature; and those that are generally non-altitude-related, such as relative humidity (RH) and sunshine hours (SunH). Therefore, both altitude-related and generally non-altitude related environmental variables may play roles in driving population adaptive divergence. Since the exclusive habitats of *C. formosana* may be reduced or even disappear in view of global climate change, its unique distribution from low to high elevations makes it useful for investigating the role of elevation on genetic divergence in association with environmental conditions. Information obtained in this study can be useful in the future assisted migration program of *C. formosana*. We surveyed AFLP variation of 243 samples from 12 populations of *C. formosana* to examine the role of gene flow on the pattern of population genetic diversity and to test environmentally driven local adaptation in *C. formosana* populations. Our goals in the present study were to (i) assess whether there is a pattern of genetic diversity along elevation across forest vegetation zones; (ii) test the effect of environment relative to geography on adaptive genetic divergence; and (iii) investigate whether there are low- and high-elevation adaptive genetic loci with high frequencies that are likely to play roles in local adaptation.

## Materials and Methods

### Sampling and Genotyping

We collected fresh leaf samples of 243 *C. formosana* individuals from 12 populations distributed in Taiwan (Figure 1 and Table 1). Total DNA was extracted from ground-up leaf powder according to a modified cetyltrimethyl ammonium bromide (CTAB) procedure (Doyle and Doyle, 1987). Ethanol precipitated DNA was washed with 70% ethanol, dissolved in 200 μL TE buffer (pH 8.0) and stored at −20°C until needed. The DNA concentration was determined for each sample using the NanoDrop spectrophotometer (NanoDrop Technology, Wilmington, DE, United States). We quantified genetic variation using AFLP (Vos et al., 1995). Digestion of total genomic DNA (100 ng) was performed using 2 U *Eco*RI and 2 U *Mse*I (New England Biolabs, Ipswich, MA, United States) and digested DNA products were added to a 10-μl reaction mixture for ligation at 22°C for 1 h. The ligation reaction mixture contains 5 μM of the *Eco*RI adaptor, 50 μM of the *Mse*I adaptor, and 0.25 U T4 DNA ligase (Real Biotech, Taipei, Taiwan). 1 μl diluted digested samples (1:9 dilution with water) was used as a template to perform pre-selective amplification. Pre-selective amplification was performed in a 10-μl volume containing 1 × PCR buffer (25 mM KCl, 10 mM Tris-HCL, 1.5 mM MgCl_2_, and 0.1% Triton X-100), 100 nM each of the *Eco*RI (E00: 5′*-*GACTGCGTACCAATTC*-*3′) and *Mse*I (M00: 5′*-*GATGAGTCCTGAGTAA*-*3*′*) primers, 0.25 mM dNTPs, and 1 U *Taq* DNA polymerase (Zymeset Biotech, Taipei, Taiwan). Conditions of the pre-selective amplification were initial holding at 72°C for 2 min and pre-denaturation at 94°C for 3 min, followed by 25 cycles of 30 s at 94°C, 30 s at 56°C, and 1 min at 72°C, with a final 5-min holding at 72°C. Eleven *Eco*RI-*Mse*I selective primers combinations with sequences of E00 and M00 and additional bases were added at the ends of these primers (Supplementary Table 1). In selective amplification, *Eco*RI selective primer was labeled with fluorescent dye (6-FAM or HEX). Conditions of selective amplification were a 10-μl volume containing 1 × PCR buffer, 100 nM of the *Eco*RI selective primer, 100 nM of the *Mse*I selective primer, 0.25 mM dNTPs, 0.75 U *Taq* DNA polymerase, and 1 μl diluted pre-selective amplified product (1:9 dilution with water). The selective PCR amplification was performed with initial holding at 94°C for 3 min, followed by 13 cycles of 30 s at 94°C, 30 s at 65°C with 0.7°C touchdown per cycle, and 1 min at 72°C, then 23 cycles of 30 s at 94°C, 30 s at 56°C, and 1 min at 72°C, with a final 5-min holding at 72°C. PCR amplified fragments were separated using an ABI 3730XL DNA analyzer and scored with Peak Scanner v.1.0 (Applied Biosystem, Foster City, CA, United States). We scored AFLP fragments with a fluorescent threshold set at 150 units. Peaks of amplified fragments in the range of 150–500 bp separated by less than one nucleotide in a ±0.8 base pair window were scored as the same fragment. We removed markers scored higher than 99% or less than 1% of individuals. Locus genotyping error rate of each primer combination was calculated based on amplification replicates obtained from three samples in each population. Loci with error rate greater than 5% were removed (Bonin et al., 2004). The mean error rate was 4.91% (Supplementary Table 1). The final AFLP dataset was deposited in Supplementary Data Sheet 1.

### Genetic Diversity and Clustering

Proportion of polymorphic loci (%*P*, 95% criterion) and unbiased expected heterozygosity (*uH*_E_) within a population (Nei, 1987) were calculated using AFLP-SURV v.1.0 (Vekemans et al., 2002) based on allele frequencies estimated assuming Hardy-Weinberg equilibrium with non-uniform prior distribution (Zhivotovsky, 1999). Per locus *uH*_E_ was calculated using ARLEQUIN v.6.0 (Excoffier and Lischer, 2010). Index of association (*I*_A_) (Brown et al., 1980) and modified index of association (*r*D) (Agapow and Burt, 2001), representing multilocus linkage disequilibrium (LD), were calculated using the *ia* function of R poppr package v.2.8.5 (Kamvar et al., 2015) in the R environment (R Development Core Team, 2018), and significant departure from zero was tested with 999 permutations. The *f*-free model of a Bayesian approach for dominant marker implemented in HICKORY (Holsinger and Lewis, 2003) was used to estimate inbreeding coefficient (*F*_IS_). Assessment of significant difference of mean *uH*_E_ per locus between populations was performed using a linear mixed model (LMM), based on reduced maximum likelihood estimation, with population as a fixed effect and locus as a random effect using the *lmer* function of R lme4 package v.1.1-21 (Bates et al., 2015). Significance was assessed based on type II Wald χ^2^ test using the *Anova* function of R car package v.3.0-6 (Fox and Weisberg, 2011) with the Tukey method to maintain α = 0.05 using the *lsmeans* function of R emmeans package v.1.4.3 (Lenth, 2018).

Sparse non-negative factorization (sNMF) algorithm (Frichot and Francois, 2015) and discriminant analysis of principal components (DAPC) (Jombart et al., 2010) were used to assess genetic homogeneous groups of *C. formosana* individuals. We assessed individual assignments with *K* = 1–12 based on least-squares optimization using the *snmf* function of R LEA package v.3.0.0 (Frichot and Francois, 2015). In *snmf*, the regularization parameter, iterations, and repetitions in *snmf* were set to 100, 200, and 10, respectively, and other arguments set to defaults. In DAPC, principal component analysis was performed and followed by a discriminant analysis that maximize variance between groups using the *find.clusters* and *dapc* functions of R adegenet package v.2.1.2 (Jombart and Ahmed, 2011). The best *K* in LEA and DAPC was, respectively, evaluated with the mean of minimal cross-entropy (CE) and the Bayesian information criterion (BIC).

### Environmental Heterogeneity

We acquired 34 environmental variables and assigned to three categories including 19 bioclimatic, three topological, and 12 ecological variables. Nineteen bioclimatic variables, constituting weather station-based temperature and precipitation information, for sample sites at 30-s spatial resolution (∼1 km) were downloaded from the WorldClim v.1.4 (Hijmans et al., 2005). Topographic variables at 30-m resolution, including aspect, elevation, and slope, were obtained from Aster Global Digital Elevation Map^1^. Ecological factors obtained were normalized difference vegetation index (NDVI) and enhanced vegetation index (EVI) derived from moderate resolution imaging spectroradiometer (MODIS) dataset MOD13A2 (1 km resolution) and leaf area index (LAI) and fraction of absorbed photosynthetically active radiation (fPAR) derived from a MOD15A2 dataset (500 m resolution). These MODIS datasets were acquired from Land Process Distributed Active Archive Center^2^ during 2001–2018, and monthly mean values computed using a maximum-value composite procedure (Huete et al., 2002). The annual total potential evapotranspiration (PET) was also included as an ecological variable and calculated based on a MOD16A3 dataset (500 m resolution). Monthly mean values of the other five ecological variables, including RH, cloud cover (CLO), SunH, number of rainfall days per year (RainD), and mean wind speed (WSmean), were calculated with data obtained from the Data Bank for Atmospheric & Hydrologic Research (^3^ recorded in 1990–2018) at spatial resolution of 1 km using a universal spherical model of the Kriging method in ArcGIS (Chang et al., 2014). Soil pH values of sample sites were obtained based on an island-wide soil investigation (*n* = 1150) conducted in 1969–1986 obtained from the Agriculture and Food Agency of Taiwan (Chang et al., 2009). Annual moisture index (Thornthwaite, 1948) was calculated based on annual potential evapotranspiration derived from annual mean temperature and annual precipitation.

We used the *cor* function of R to calculate correlation coefficient (*r*) between environmental variables. The *vif* function of R package usdm v.1.1-18 (Naimi et al., 2014) was used to calculate variance inflation factor (VIF) of environmental variables for each environmental category (bioclimate, ecology, and topology) separately. Environmental variables within each category with VIF < 5 and which not strongly correlated with other variables (|*r*| < 0.8) were retained. Fourteen environmental variables retained were: BIO1 (annual mean temperature), BIO7 (annual temperature range), BIO12 (annual precipitation), and BIO18 (precipitation of the warmest quarter) of bioclimatic category; aspect, elevation, and slope of topological category; and fPAR, NDVI, RH, RainD, soil pH, sunH, and WSmean of ecological category (Supplementary Table 2). The matrix of pairwise correlation coefficient between these environmental variables was used to depict correlation plot using the *corrplot* function of R package corrplot v.0.84 (Wei and Simko, 2017).

To assess environmental differences among sample sites and among sample sites of the three genetic clusters (see section “Results”), Euclidean distance matrix was generated using the *dist* function of R for the 14 retained environmental variables and used in a permutational multivariate analysis of variance (PERMANOVA) using *adonis* function of R package vegan v.2.5-6 (Oksanen et al., 2017). Pairwise comparison was performed using the *pairwise.perm.manova* function of R package RVAideMemoire v.0.9-74 (Herve, 2018). Significance of pairwise comparisons was tested with 999 permutations and a false discovery rate (FDR) of 5%.

### Test for *F*_ST_ Outliers

Two *F*_ST_-based methods (BAYESCAN and DFDIST) were used to identify *F*_ST_ outliers indicating signature of selection across populations. BAYESCAN v.2.1 (Foll and Gaggiotti, 2008) uses a reversible-jump Markov chain Monte Carlo algorithm to estimate the ratio of posterior probabilities of selection over neutrality [the posterior odds (PO)] using hierarchical Bayesian method. Two hundred pilot runs of 50,000 iterations were performed followed by a sample size of 50,000 with thinning interval of 20 among 10^6^ iterations in BAYESCAN analysis. A logarithmic scale of log_10_PO > 0.5, of log_10_PO > 1.0, of log_10_PO > 1.5, and of log_10_PO > 2 was, respectively, defined as substantial, strong, very strong, and decisive evidence for selection over neutrality according to Jeffreys (1961) and Foll (2012). In the present study, we considered a locus with log_10_PO > 2 as a potential selective outlier.

The Beaumont and Nichols model, modified for dominant marker, implemented in DFDIST (Beaumont and Nichols, 1996) was used to estimate a distribution of observed *F*_ST_ (Weir and Cockerham, 1984) versus *uH*_E_ (Zhivotovsky, 1999). Loci that may be under selection were identified by comparing to a simulated neutral distribution. Parameters include critical frequency = 0.99; Zhivotovsky parameters = 0.25; trimmed mean *F*_ST_ = 0.3 (excluding 30% of highest and 30% of lowest *F*_ST_ values); smoothing proportion = 0.04; 500,000 resamplings; critical *P* = 0.05, and target average θ (level of differentiation) = 0.099575 were used for running DFDIST. In DFDIST, significant *P* values of *F*_ST_ outliers were evaluated at the 99% confidence level with 5% FDR.

### Test for Associations of Genetic Loci With Environmental Variables

Samβada v.0.8.3 (Stucki et al., 2017) and latent factor mixed model (LFMM) implemented in the *lfmm* function of R LEA package (Frichot et al., 2013) were used to assess the associations of all the genetic loci with environmental variables. Samβada employs a multiple univariate logistic regression approach to assess the significant correlations of allele frequencies with the values of environmental variables. Both Wald and G scores with an FDR cutoff of 0.01 were applied to assess the significant fit of model with environmental variables compared to null model without environmental variables. LFMM uses a hierarchical Bayesian mixed model considering background level of population structure, latent factors (*K*), as a random effect due to demographic history and IBD pattern. Genetic data was used as a fixed effect in a testing procedure based on *Z*-scores. The number of latent factors, *K*, was set to 3 based on the results of LEA and DAPC analyses (see section “Results”). Five LFMM runs for each value of *K* with 10,000 iterations of the Gibbs sampling algorithm and a burn-in period of 5,000 cycles were performed. Z-scores of five independent runs were combined using Fisher-Stouffer method (Brown, 1975), and the resulting *P*-values were adjusted using the genomic inflation factor (λ). An FDR correction of 1% was further used in *P*-value adjustment using the *q-*Value function of *R q*-value package v.2.20.0 (Storey et al., 2019). AFLP loci recognized as outliers potentially evolved under selection were those *F*_ST_ outliers detected either by BAYESCAN or by DFDIST and found to be strongly associated with environmental variables.

We used a Bayesian logistic regression analysis implemented in the *stan_glm* function of R rstanarm package v.2.19.2 (Goodrich et al., 2018) to further verify the associations of *F*_ST_ outliers detected by BAYESCAN and DFDIST with environmental variables. Student’s *t* distribution with mean zero and seven degrees of freedom were used as the weakly informative priors, and the scale of the prior distribution was 10 for the intercept and 2.5 for the predictors. All *stan_glm* models were run with four chains for 2000 warm-up and 2000 sampling steps. The *posterior_interval* function of R rstanarm package was employed to estimate 95%, 99%, and 99.5% credible intervals for determination of significant correlations of *F*_ST_ outliers with environmental variables.

### Genetic Differentiation

Both the total and the outlier data were used in analysis of molecular variance (AMOVA). AMOVA was used to estimate the levels of population genetic differentiation at the hierarchical levels of geographic areas north and south of the HMR, genetic clusters (see section “Results”), and sample sites using the *poppr.amova* function of R package poppr, and significance tested using the *randtest* function of R package ade4 v.1.7-13 (Dray and Dufour, 2007) with 9,999 permutations. Pairwise *F*_ST_ was computed using ARLEQUIN based on the total data and significance tested with 10,000 permutations.

### Mantel Test, Variation Partitioning, and Forward Selection

Mantel test was used to analyze the correlations of the AFLP Euclidean distance matrix with the Euclidean distance matrix of geography (latitude and longitude) and with the matrix of elevational difference using the *mantel* function of R vegan package. The retained environmental variables in the three environmental categories (bioclimatic, ecological, and topological categories) were analyzed separately using redundancy analysis (RDA) to assess the relative contribution of environmental variables in each category explaining the outlier AFLP variation using the *varpart* function of R package vegan, and significance tested using the *anova.cca* function with 999 permutations. Four fractions of the total outlier variation were partitioned: pure environmental variables (fraction [a]), geographically structured environmental variables (fraction [b]), pure geographic variables (fraction [c]), and residual effects (fraction [d]) (Borcard et al., 1992; Borcard and Legendre, 2002). The amount of the outlier variation explained in each fraction was determined based on adjusted *R*^2^ values (Peres-Neto et al., 2006). Longitude and latitude of sample sites were used as geographic variables in the analysis. The double-stopping criterion (Blanchet et al., 2008) of the *forward.sel* function of R adespatial package v.0.3-7 (Dray et al., 2019) was used to estimate the most important environmental variables explaining the outlier genetic variation and significance determined using 999 permutations.

## Results

### Diversity

We obtained 437 AFLP (mean ± SD: 39.7 ± 12.0) loci using 11 selective amplification primer combinations (Supplementary Table 1). The average percentage of polymorphism was 42.1 (ranged from 36.6 for the population FCH to 50.8 for the population CL) (Table 1). The average level of *uH*_E_ was 0.146 and ranged between 0.104 (population FCH) and 0.175 (population KW). No positive correlation between population *uH*_E_ and sample size was found based on Spearman’s rank correlation test (ρ = -0.371, *P* = 0.237). The measures of multilocus LD, *I*_A_ and *r*D, showed significant departure from random association between AFLP loci for all populations except the population SS (Table 1). Moreover, population *F*_IS_ estimated using the *f*-free model of HICKORY ranged between 0.497 and 0.509.

Within *C. formosana*, LMM analysis showed the level of mean *uH*_E_ per locus was significantly different among populations (χ^2^ = 164.24, *P* < 0.0001). Significant difference was also found in many pairwise population comparisons (Supplementary Table 3), in which paired populations in comparisons were found not only located in the same, but also in different forest vegetation zones.

### Genetic Structure

**FIGURE 2:**
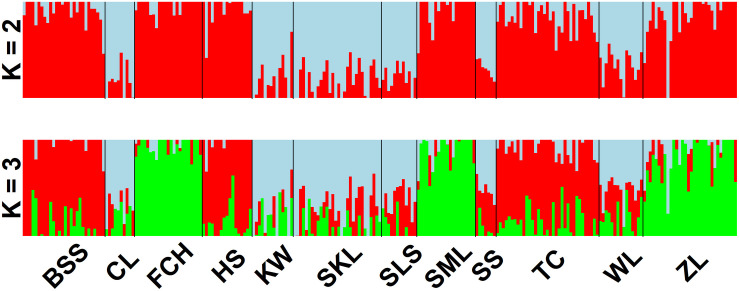
Individual assignments of 243 individuals from 12 populations of *Calocedrus formosana* analyzed using LEA. The clustering scenarios for *K* = 2–3 were displayed.

**FIGURE 3:**
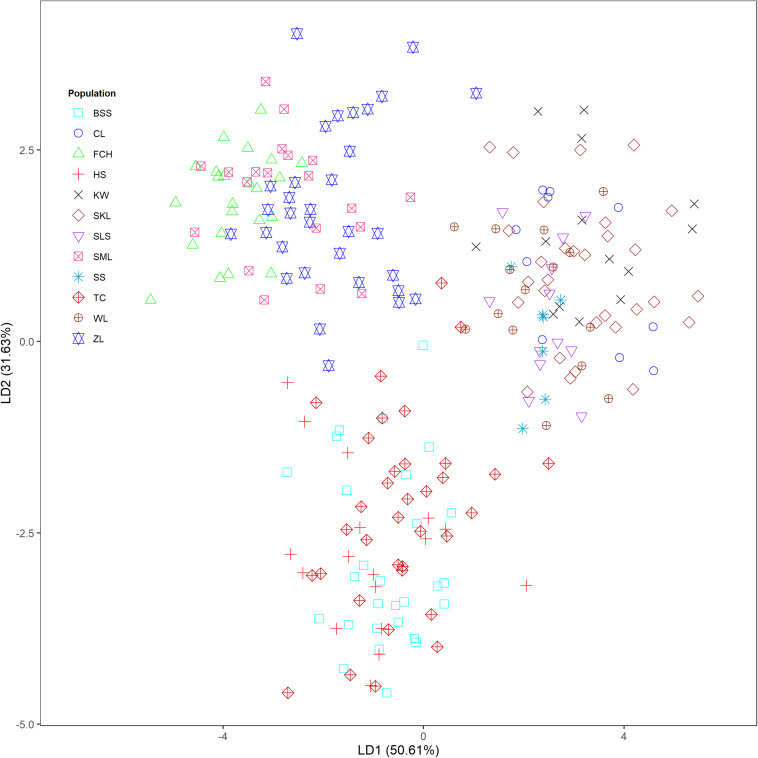
Clustering results analyzed using discriminant analysis of principal components (DAPC) for the 243 individuals from 12 populations of *Calocedrus formosana*.

The mean of minimal CE was minimized after *K* = 4 in LEA analysis (Supplementary Figure 1A) and the BIC in DAPC analysis was minimized at *K* = 5. (Supplementary Figure 1B). However, three genetically homogeneous groups were most reasonably displayed by both LEA and DAPC (Figures 2, 3). The amount of genetic variation explained by the first two linear discriminants was, 50.61% and 31.63%, respectively (Figure 3). The first cluster contains populations WL, SS, CL, SKL, KW, and SLS. The second cluster contains populations TC, BSS, and HS. Populations SML, FCH, and ZL were included in the third genetic cluster. Using the total data, AMOVA revealed significant differentiation at all hierarchical structuring levels analyzed between populations (*Φ*_ST_ = 0.1402, *P* < 0.001), between populations of different genetic clusters (*Φ*_ST_ = 0.1283, *P* < 0.001), and between north and south of the HMR (*Φ*_ST_ = 0.0967, *P* < 0.001) (Table 2). However, the level of population differentiation between north and south of the HMR was relatively low compared to across population differentiation, albeit significant. The average pairwise *F*_ST_ was 0.1327 based on the total data (Supplementary Table 4).

**TABLE 2:** Summary of the analysis of molecular variance (AMOVA) based on the total and the outlier genetic variations.

**Source of variation**	**Data**	**df**	**Sum of squares**	**Percent of variation**	**Φ Statistics**	***P-*value**
Between populations	Total	11	1293.374	14.02	*Φ*_ST_ = 0.1402	<0.001
	Outlier	11	476.7049	34.48	*Φ*_ST_ = 0.3448	<0.001
Within populations	Total	231	6399.005	85.98		
	Outlier	231	872.6733	65.52		
Total	Total	242	7692.379	100		
	Outlier	242	1349.3827	100		
Between genetic clusters	Total	2	744.507	12.83	*Φ*_ST_ = 0.1283	<0.001
	Outlier	2	365.1407	35.00	*Φ*_ST_ = 0.3499	<0.001
Within genetic cluster	Total	240	6947.871	87.17		
	Outlier	240	984.2420	65.00		
Total	Total	242	7692.379	100 100		
	Outlier	242	1349.3827	100		
Between north and south of the HMR	Total	1	370.182	9.67	*Φ*_ST_ = 0.0967	<0.001
	Outlier		162.7928	23.49	*Φ*_ST_ = 0.2349	<0.001
Within north and south of the HMR	Total	241	7322.197	90.33		
	Outlier		1186.5899	76.51		
Total	Total	242	7692.379	100		
	Outlier		1349.3827	100		

*HMR, Hsuehshan mountain range. Three genetic clusters distinguished by the LEA and DAPC were cluster 1: populations WL, SS, CL, SKL, KW, and SLS; cluster 2: populations TC, BSS, and HS; and cluster 3: populations SML, FCH, and ZL.*

### Potential Selective Outliers and Outlier Genetic Differentiation

BAYESCAN identified 26 loci as potential selective outliers (Table 3). Nine loci were identified as potential outliers by DFDIST; however, none of these loci remained significant after FDR correction. All 26 outliers identified by BAYESCAN were found to be strongly associated with environmental variables assessed using Samβada and LFMM (Table 3). The corresponding *Z*-scores and *q* values of candidate outlier loci identified using LFMM were summarized in Supplementary Table 5. The associations of potential selective outlier loci with more than one environmental variable were commonly observed, and therefore, Bayesian logistic regression was further used to assess the relationships between those outliers with environmental variables. Analysis using Bayesian logistic regression confirmed the associations of outlier loci with more than one variable within each environmental category (i.e., bioclimate, ecology, and topology) (Table 3).

**TABLE 3:** Potential outliers identified by BAYESCAN and DFDIST associated with environmental variables.

**Locus**	**BAYESCAN**	**Association with environmental variables**													
	**log_10_ (PO)**														

		**Bioclimate**				**Topology**			**Ecology**						
		**BIO1**	**BIO7**	**BIO12**	**BIO18**	**Aspect**	**Elevation**	**Slope**	**fPAR**	**NDVI**	**RainD**	**RH**	**Soil pH**	**SunH**	**WSmean**
aP11_1650	1000	a,B,***	b	b	a,B,***		a,B	a,***	B			a,B,***		a,B	a,b
aP11_1984	6	*	*		B,***			***	B		***	B,***	b	B,***	***
aP11_2003	2.8713	*		b	a,b,***			***	b,*	**		a,B,***	*		a
aP11_2338	5.5229	***			a,b,***			*	B		***	a,B,***		b,*	a,*
aP11_4330	1000	a,***			a,b,***	***	B		B		*	a,B,***			a,b
aP24_1967	2.0054	a,B,***	b,*				a,B,***			a,B				a,B,*	
aP24_3746	3.8664	B,*			b,**	a,***	b	a,B,***	B			a,B,***	b	b,*	a,b
aP34_1606	1000	***	a,B,***	b	b				B		***	a,B,***		a,B	***
aP34_1681	2.501	a,B,***	b,*		*		a,b,***	*		a,***		***	*	a,B	
aP34_1762	3.1609	a,B,***	B	b		*	a,B,**	a,B,***	b		***	a,B		***	a,B,***
aP34_2113	1000	a,B,***	a,b,***	b	b	*			B,*	a,***		a,B,***	b,*	a	**
aP34_2507	1000	a,***	a,B,*	a,b			*	*	a,**	a,*	b	a,B,***	b,***	a,B,***	a,b
aP34_2811	1000	*	a	b					b,*	*	b	a,b,**	b,***	a,B,***	
aP34_3008	2.0686	a,***				b,***	a,B,***		b,*	a,***	b,*		*		
aP35_2216	3.0748		B,***		***	b	b	**	b		b,***	**	a,B,***	a,B,***	
aP38_2271	1000	a,***			**	*	a,B,*	a	B			a,B,***		*	a,b,***
aP47_3520	1000	a,B,***	***			***	a,B,***		*	a,***		***	b,**	a,b	*
aP49_1747	1000	B,***			b,***	a,***	b		a,B		*	a.b,***	b		a,b
aP49_2083	1000	***	a,b,***	a,b		*		***	a,b	a		a,**		a,B,**	
aP49_2597	5.1549	a,B,***	***	*		b,***	a,b,***	*		a,***	b	***	***		**
aP49_2698	3.1287	**	b		*				B		b,*	a,B,***			a,b
aP55_1883	1000	***	b			*	**	***	*	a		a		a,b,***	a*
aP56_1840	5.301				a,B,***			a,***		a,B	*	a,B,***	b,**	a,B,*	
aP57_1778	1000	*	b			*		*	b,***	*		*	b	a,***	b
aP57_2129	4.1426	a,B,***			b		a,B,***		b,***	***	b,*	a,b,***	***		*
aP57_3127	2.152		b					***	b,***	***		b	b	a,b	

a and b represent significant correlation of AFLP markers with individual environmental variables identified by Samβada and LFMM, respectively. B represents a |Z| ≥ 1.5 in LFMM analysis. *,**,*** significance based on 95, 99, and 99.5% posterior credible intervals for the potential outliers found to have strongly correlated with specific environmental variable(s) using the stan_glm function of R package rstanarm. Aspect (0–360°) and slope (0–90°). BIO1, annual mean temperature; BIO7, annual temperature range; BIO12, annual precipitation; BIO18, precipitation of the warmest quarter; fPAR, fraction of absorbed photosynthetically active radiation; NDVI, normalized difference vegetation index, RainD, number of rainfall days per year; RH, relative humidity; SunH, sunshine hours; WSmean, mean wind speed.

**FIGURE 4:**
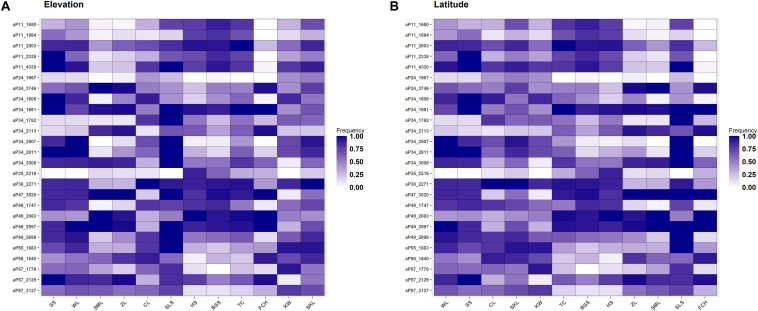
Heatmap of allele frequencies of the 34 outlier loci identified. The sequence of populations was arranged according to **(A)** elevation or **(B)** latitude.

Allele frequencies of the outlier loci across populations arranged altitudinally were depicted in Figure 4A. The most prominent outlier AFLP loci with high frequencies uniquely at low elevations were aP11_2338 (population SS) and aP34_1606 (population SS). At high elevations, aP57_1778 (population KW) was the most prominent outlier AFLP locus. Additionally, there were also outlier AFLP loci, e.g., aP34_3008 (population SLS), aP35_2216 (population HS), and aP49_2698 (population SLS), found to have high frequencies at middle elevations between 800 and 1400 m in the montane evergreen cloud zone. Medium frequency outlier AFLP loci were also commonly observed at different elevational ranges corresponding to different forest vegetation zones.

Genetic differentiation analyzed using AMOVA, based on the outlier data, showed significant outlier genetic differentiation between populations (*Φ*_ST_ = 0.3448, *P* < 0.001; Table 2), between populations of the three genetic clusters (*Φ*_ST_ = 0.3499, *P* < 0.001), and between populations north and south of the HMR (*Φ*_ST_ = 0.2349, *P* < 0.001).

### Outlier Genetic Variation Explained by Environment and Geography and the Most Important Environmental Variables Associated With the Outlier Variation

The correlation between the 14 retained environmental variables was depicted in Supplementary Figure 2. Although PERMANOVA revealed no environmental difference across populations (*P* = 1), significant environmental differences between sample sites and between sample sites of different vegetation zones were found (Supplementary Table 6). Additionally, environmental heterogeneity was found when compared among the sample sites grouped into three genetic clusters (*F* = 23.01, *R*^2^ = 0.1609, *P* = 0.001). Mantel test revealed significant IBD based on the total and the outlier data (total data: Mantel *R*^2^ = 0.185, *P* = 0.0001; outlier data: Mantel *R*^2^ = 0.348, *P* = 0.0001). Total explainable outlier genetic variation was 18.52%, 30.09%, and 19.64%, respectively, for bioclimatic, ecological, and topological variables analyzed separately (Table 4). In all three environmental categories, the amount of explained variation was relatively small as compared to the amount of unexplained variation (fraction [d]). Based on the outlier genetic data and analyzed the three environmental categories separately, pure ecological factor was found to be the most influential environmental variables contributing to the outlier genetic variation (18.75%) in contrast to pure bioclimatic (7.18%) and pure topological (8.30%) factors. Within bioclimatic and within ecological category, pure environmental factor explained much larger proportion of the outlier genetic variation than those explained by pure geography (7.18% vs. 4.26%; 18.75% vs. 4.45%, respectively). However, pure geography (11.84%) explained a larger fraction of the outlier variation than pure topological factors (8.30%) within the topological category. Additionally, outlier genetic variation can also be explained by geographically structured environmental variables in bioclimatic and in ecological category (7.07% and 6.89%, respectively), but no outlier genetic variation was explained by geographically structured environmental variables in topological category.

**TABLE 4:** The percentage of the outlier genetic variation accounted for by non-geographically structured environmental variables [a], shared (geographically structured) environmental variables [b], pure geographic factors [c], and undetermined component [d] analyzed based on the 14 retained environmental variables in three environmental categories (bioclimate, ecology, and topology).

	**Bioclimate**			**Ecology**			**Topology**		
	**Adjusted *R*^2^**	***F***	***P***	**Adjusted *R*^2^**	***F***	***P***	**Adjusted *R*^2^**	***F***	***P***
AFLP									
[a]	0.0718	6.29	0.001	0.1875	10.20	0.001	0.0830	9.78	0.001
[b]	0.0707	–	–	0.0689	–	–	−0.0050	–	–
[c]	0.0426	7.23	0.001	0.0445	8.47	0.001	0.1184	14.81	0.001
[a + b + c]	0.1852	10.17	0.001	0.3009	12.57	0.001	0.1964	9.78	0.001
[d]	0.8148		0.6991	–	–	0.8036	–	–	

*Proportions of explained variation were obtained from variation partitioning by redundant analysis. *F* and *P*-values are specified for testable fractions. Fraction [b] is untestable because the adjusted R^2^ value was obtained by subtraction ([a + b + c]-[a]-[c]) rather than by estimation.*

We assessed the most important variables in each environmental category accounted for the outlier genetic variation using forward selection (Blanchet et al., 2008). The most significant influential environmental variable was annual mean temperature (bioclimate), relative humidity (ecology), and elevation (topology) (adjusted *R*^2^ = 5.67%, adjusted *R*^2^ = 13.69%, and adjusted *R*^2^ = 3.55%, respectively) based on the outlier data (Table 5).

**TABLE 5:** Environmental variables selected by a forward selective procedure explaining outlier genetic variation in *Calocedrus formosana.*

	**Environmental Variables**	**Adjusted *R*^2^**	**Cumulative adjusted *R*^2^**	***F* statistic**	***P-*value**
Bioclimate	BIO1	0.0567	0.0567	15.56	0.001
	BIO7	0.0562	0.1129	16.25	0.001
	BIO18	0.0281	0.1410	8.85	0.001
Ecology	RH	0.1369	0.1369	39.39	0.001
	SunH	0.0780	0.2176	24.94	0.001
	Soil pH	0.0093	0.2269	3.93	0.001
	RainD	0.0080	0.2349	3.37	0.001
	fPAR	0.0079	0.2428	3.46	0.001
	NDVI	0.0076	0.2504	3.92	0.001
	WSmean	0.0072	0.2576	3.30	0.001
Topology	Elevation	0.0355	0.0355	9.91	0.001
	Slope	0.0257	0.0612	7.60	0.001
	Aspect	0.0168	0.0780	5.37	0.001

*BIO1, annual mean temperature; BIO7, annual temperature range; BIO18, precipitation of the warmest quarter; fPAR, fraction of absorbed photosynthetically active radiation; NDVI, normalized difference vegetation index, RainD, number of rainfall days per year; RH, relative humidity; SunH, sunshine hours.*

## Discussion

### Patterns of Diversity and the Roles of Gene Flow and Environments on Population Diversification

Population genetic diversity is the basis for evolution in a species that can result from the balance between gains of new mutations and drift-mediated loss of alleles (Kimura and Crow, 1964; Barrett and Schluter, 2008; Jump et al., 2009). Plants that occupy restricted geographic range are expected to have reduced level of genetic diversity (Hamrick and Godt, 1989; Cole, 2003). However, *C. formosana* distributed in a wider elevational range lower level of AFLP genetic diversity (average *uH*_E_ = 0.146) was found compared with other Taiwanese conifers with narrower geographic distributions such as *K. davidiana* var. *formosana* (average *uH*_E_ = 0.233, Fang et al., 2013), *C. konishii* (*uH*_E_ = 0.203, Li et al., 2019), and *T. cryptomerioides* (*uH*_E_ = 0.236; Li et al., 2018).

Genetic diversity is greater for central than for marginal populations because conditions at the central portion of the range are optimal, but environments at the edges can be stringent (Lesica and Allendorf, 1995; Eckert et al., 2008). Although migration can contribute intra-population genetic diversity (Maruyama, 1970; Nei, 1973; Barrett and Schluter, 2008; Jump et al., 2009), populations located at the outer boundaries of the species’ range genetic drift can be promoted, thereby reducing genetic diversity and increasing differentiation from central populations (Kimura and Crow, 1964; Maruyama, 1970; Nei, 1973; Barrett and Schluter, 2008; Jump et al., 2009). Our results of the level of *uH*_E_ along elevational gradient in the present study are not consistent with the abundant-center hypothesis and higher levels of *uH*_E_ can be found in the outer margin (low- and high- elevations) populations compared with that of central populations (c.f. Assis et al., 2013; Figure 1 and Table 2).

The positive *F*_IS_ and significant multilocus LD can result from bottlenecks because of mating among genetically close individuals within populations (Przeworski, 2002; Wall et al., 2002). Past bottleneck events can effectively cause reduction in sample size, loss of connectivity, increase in genetic drift, and increase in the probability of inbreeding (Reed and Frankham, 2003). The current reproductive mode of inbreeding is evident in *C. formosana* populations because of significant positive *F*_IS_ values estimated for all populations and significant departure from random association based on the measures of multilocus LD in all but one of the populations examined (*I*_A_ and *r*_D_, Table 1). The non-random breeding or mating between genetically close relatives of a population could be a general phenomenon in Taiwanese conifers because significant positive *F*_IS_ and significant departure from random association are also found in other Taiwanese conifers such as *C. konishii*, *K. davidiana* var. *formosana*, and *T. cryptomerioides* (Fang et al., 2013; Li et al., 2018, 2019). Moreover, the magnitude of loss of alleles might be greater in *C. formosana* populations compared with the other three Taiwanese conifers. Nonetheless, divergent selection invoked by environmental heterogeneity, resulting in the higher level of population differentiation compared with other Taiwanese conifers (Fang et al., 2013; Li et al., 2018, 2019), might have also contributed to the detection of significant multilocus LD (Kawecki and Ebert, 2004; Nosil et al., 2005; Jump and Peñuelas, 2006).

Efficient migration can be the cause for the low level of differentiation between subdivided populations (Maruyama, 1970; Nei, 1973) and the general realization in conifers is the high rates of effective pollen dispersal, resulting in low population differentiation (Hamrick et al., 1992; Hamrick and Godt, 1996). Relatively higher level of the average pairwise *F*_ST_ was found in *C. formosana* (=0.1327) based on the total data in the present study (Supplementary Table 4) compared to other Taiwanese conifers such as *T. cryptomerioides* (average pairwise *F*_ST_ = 0.041, Li et al., 2018), *C. konishii* (average pairwise *F*_ST_ = 0.102, Li et al., 2019), and *K. davidiana* var. *formosana* (average pairwise *F*_ST_ = 0.061, Fang et al., 2013). AMOVA also showed higher level of across population differentiation based on the total data in *C. formosana* (*Φ*_ST_ = 0.1647) compared to *T. cryptomerioides* (*Φ*_ST_ = 0.0316, Li et al., 2018), *C. konishii* (*Φ*_ST_ = 0.0827, Li et al., 2019), and *K. davidiana* var. *formosana* (*Φ*_ST_ = 0.0632, Fang et al., 2013) occurring in Taiwan. The higher level of population differentiation in *C. formosana* might have been caused by a relatively lower rate of gene flow, resulting in larger degree of drift-mediated loss of alleles (Kimura and Crow, 1964; Conner and Hartl, 2004; Barrett and Schluter, 2008; Jump et al., 2009).

Nonetheless, environments may also play roles in shaping population differentiation (Kawecki and Ebert, 2004; Nosil et al., 2005; Jump and Peñuelas, 2006; Holderegger et al., 2008; Jump et al., 2009; Table 2). PERMANOVA revealed differences in environmental conditions between sample sites and between sample sites of different vegetation zones (Supplementary Table 6), suggesting that environmental heterogeneity between sample sites and between sample sites located in different vegetation zones might have played roles in driving population differentiation along elevational gradients. In the present study, Mantel test revealed significant IBD based on the total and the outlier data. Moreover, mountains might not have played a significant role as barriers to gene flow between north and south of the HMR because AMOVA showed a lower level of differentiation based on the total data (*Φ*_ST_ = 0.0967, Table 2) for populations located between these two geographic areas compared to the higher level of across population differentiation (*Φ*_ST_ = 0.1402). The HMR may not act as an effective barrier to gene flow can also be supported by the grouping of population SLS located in the south of the HMR with populations WL, SS, CL, SKL, and KW located in the north of the HMR in the same genetic cluster revealed by the LEA and DAPC analyses (Figures 1, 2).

Evolutionary divergence can occur in the presence of gene flow (Wu, 2001) and the magnitude of migration might have a relationship with the probability of detecting adaptive divergence in these Taiwanese conifers. The number of environmentally dependent selective outliers among the four Taiwanese conifers mentioned might have a positive relationship with the level of population differentiation. The number of environmentally dependent selective outliers out of the total AFLP loci scored are 0/1413 (0%, *T. cryptomerioides*, Li et al., 2018), 3/465 (0.65%, *K. davidiana* var. *formosana*, Fang et al., 2013), 15/482 (3.11%, *C. konishii*, Li et al., 2019), and 26/483 (5.95%, *C. formosana*, Table 3). It is worthy to note that more *F*_ST_ outliers were detected in the present study even a more stringent criterion (log_10_(PO) > 2) for outlier identification was employed in BAYESCAN as compared to the criterion of log_10_(PO) > 0.5 used in the other three previous studies.

In general, we can summarize a pattern of lower level of genetic diversity along with the higher level of genetic differentiation for Taiwanese conifers including *C. konishii*, *K. davidiana* var. *formosana*, *T. cryptomerioides*, and *C. formosana*. The relationship of higher number of environmentally dependent selective outliers detected with higher levels of genetic differentiation in these conifers suggests that gene flow among environmentally distinct populations can be effective in homogenizing locally adapted alleles and dampened local adaptation driven by natural selection (Kirkpatrick and Barton, 1997; Lenormand, 2002; Sexton et al., 2014).

### Ecological Factors Played the Most Prominent Roles in Driving Adaptive Evolution

Geography and environment can both be effective barriers to gene flow between populations (Hahn et al., 2012; Wang et al., 2013; Sexton et al., 2014; Reis et al., 2015; Antonelli, 2017; Feijó et al., 2019). These factors are not mutually exclusive and they are closely related directly or indirectly influencing the process of dispersal (Wang et al., 2013; Huang et al., 2016), and we are expecting to find both geography and environment explaining the outlier genetic variation to a certain degree. Variation partitioning controlling for geographic distance revealed significant amounts of the outlier genetic variation explained by variables in all three environmental categories (Table 4). Interestingly, ecological factors explained a relatively larger amount of the outlier variation compared to the amount of the outlier variation explained by bioclimatic factors and explained by topological factors in separate analyses. Various environmental conditions can lead to fitness-related adaptation to local conditions (Storz, 2010), reflecting in the level of differentiation as revealed by AMOVA based on environmentally dependent outlier data (*Φ*_ST_ = 0.3448, Table 2). Additionally, forward selection found that the most important variable in three environmental categories explaining the outlier genetic variation, respectively, was annual mean temperature, relative humidity, and elevation (Table 5). However, environmental variables in three environmental categories explaining more than 5% of the outlier genetic variation were annual mean temperature and annual temperature range (bioclimatic variable, adjusted *R*^2^ = 0.0567 and adjusted *R*^2^ = 0.0562, respectively) and relative humidity and sunshine hours (ecological variable, adjusted *R*^2^ = 0.1369 and adjusted *R*^2^ = 0.0780, respectively).

Temperature can play a significant role in shaping genetic divergence and enhancing speciation rate (Allen et al., 2006; Strasburg et al., 2012). It is commonly found that temperature plays a prominent role as a selective driver for adaptive divergence in various plant species (Manel et al., 2010, 2012; Bothwell et al., 2013). Temperature was also found to be one of critical environmental variables, in plant species occurring in Taiwan, strongly associated with genetic variation analyzed using expressed sequence tag simple sequence repeats (EST-SSRs) (Hsieh et al., 2013) and AFLP and methylation-sensitive amplification polymorphism (Huang et al., 2015b) in *Rhododendron oldhamii*. Temperature was also found to be strongly associated with AFLP variation in *K. davidiana* var. *formosana* (Fang et al., 2013), in *Salix fulvopubescens* (Huang et al., 2015a) and in *C. konishii* (Li et al., 2019). Association of single nucleotide polymorphism with temperature was also found in *K. davidiana* var. *formosana* (Shih et al., 2018). In *C. formosana*, annual mean temperature and annual temperature range might have played a crucial, but minor role involved in adaptive divergence (adjusted *R*^2^ = 0.0567 and adjusted *R*^2^ = 0.0562, Table 5) compared to ecological factors such as relative humidity (adjusted *R*^2^ = 0.1369, *P* = 0.001) and sunshine hours (adjusted *R*^2^ = 0.0780). Using a Mantel test, significant isolation by elevation was found based on the total (Mantel *R*^2^ = 0.257, *P* = 0.001) and the outlier (Mantel *R*^2^ = 0.289, *P* = 0.001) genetic data. However, elevational difference in meters cannot be the causal factor in driving population adaptive divergence (Körner, 2007) and elevation in topological category explained a relatively smaller fraction of the outlier variation (adjusted *R*^2^ = 0.0355) compared with annual mean temperature and annual temperature range in bioclimatic category, and relative humidity and sunshine hours in ecological category (Table 5). It is likely that adaptive divergence in *C. formosana* could have been invoked by the combination of environmental variables, particularly the combinatorial effect of altitude-related temperature and generally non-altitude-related relative humidity and sunshine hours. Relative humidity and sunshine hours significantly explaining the outlier genetic variation (cumulative adjusted *R*^2^ = 0.2176, Table 5) could be the most important environmental factors driving adaptive genetic variation in *C. formosana*. For plant species occurring in Taiwan, EST-SSR and AFLP variations were found to be strongly associated with relative humidity and sunshine hours in *R. oldhamii* (Hsieh et al., 2013). Relative humidity was also found to be associated with AFLP variation in *K. davidiana* var. *formosana* (Fang et al., 2013).

Variation partitioning also revealed significant amounts of the outlier genetic variation explained by geographically structured environmental conditions in bioclimatic and ecological environmental categories (Table 4), indicating that spatially shaped latent environmental variables also might have played an important role invoking adaptive genetic divergence in *C. formosana* (c.f. Nakazato et al., 2007; Huang et al., 2015a,b, 2016; Li et al., 2018, 2019).

Although aspect and slope might have only played minor roles in shaping genetic variation in *C. formosana* in the present study (Table 5), these two topological factors were found to be critical in shaping genetic variation of other plant species (e.g., Manel et al., 2003; Huang et al., 2015a; Shih et al., 2018). Aspect and slope can be important in influencing habitat microclimate (Hörsch, 2003; Bennie et al., 2008; Brousseau et al., 2015) and contributed to intra- and inter-species adaptive divergence (Manel et al., 2010, 2012; Nakazato et al., 2010; Monahan et al., 2012; Bothwell et al., 2013; Brousseau et al., 2015). Habitat-associated microclimate is also found to have a relationship with elevation (Clinton, 2003; Bell et al., 2014; Sunday et al., 2014). A single environmental factor is not always sufficient to explain local vegetation penology (Hwang et al., 2011) and it is probable that elevation, slope, and aspect in combination can have influence on tree structure and growth (Turner et al., 2001; Montoya-Pfeiffer et al., 2016; Hu et al., 2018), and increase the effects of IBE contributing to the geographic patterns of climatically and ecologically structured genetic variation.

### Adaptive Evolution at Elevational Margin Populations

The amount of genetic diversity, the strength of natural selection, and the extent of gene flow can all influence the probability of the occurrence of local adaptation (Hoffmann and Sgrò, 2011). Local adaptation can occur in association with conditions in different parts of a species’ distribution range and adaptive potential at elevational edges is particularly important for species’ contraction and expansion following climate change (Byars et al., 2007; Leimu and Fischer, 2008; Hereford, 2009; Chen et al., 2014; Rumpf et al., 2019; Zhang et al., 2019). However, central populations can also be vulnerable in the face of global climate change (Bennett et al., 2015). Our results of finding high frequency outlier loci at high- and low-elevations (Figure 4A) are in consistent with findings of evolutionary adaptation occur in leading- and trailing-edge populations of species shifting their ranges because of warming (Ackerly, 2003; Hampe and Petit, 2005; Thuiller et al., 2008; Chen et al., 2014; Rödin-Mörch et al., 2019). Additionally, outlier loci beginning to accumulate their frequencies at different elevations were also observed, suggestive of an ongoing process of outliers accumulating frequencies as selective pressures persist at local scales. In contrast, we did not find high frequency outlier loci specifically at the populations of extreme latitudinal ends of *C. formosana* distribution range (Figure 4B).

Biogeographical as well as ecological factors can affect the level of diversity within and among populations (Pérez de Paz and Caujapé-Castells, 2013). Forest upward migration of 1,500 to 1,600 m in Taiwan since the last glacial maximum has been reported (Liew and Chung, 2001) and mountain plants experienced an upward migration of ca. 3.6 m per year during the last century has also been reported (Jump et al., 2012). Nonetheless, it is common that species and genera continued to grow at the same latitude even though suitable habitats would have been reduced when forest species distributions shifted upward to a different range of elevations in response to postglacial climatic warming (Tsukada, 1966; Liew and Chung, 2001). It is probable that high elevation populations (populations KW and SKL) are recently colonized because of distribution range shifting upward, and they encountered ecological discontinuity invoking the evolution of environmentally associated genetic variation underlying local adaptation. Moreover, trailing-edge populations persisted at low elevations environmentally dependent genetic variation can also be evoked (Byars et al., 2007; Leimu and Fischer, 2008; Hereford, 2009; Chen et al., 2014; Zhang et al., 2019).

## Conclusion

For plants with limited dispersal capabilities, global climate change may manifest the risk of extinction because of loss of the unique genetic diversity at elevational margin populations. Here, our results showed no pattern of population genetic diversity in consistent with the abundant central hypothesis. Relatively lower level of genetic diversity and higher level of genetic divergence were found compared with other Taiwanese coniferous species such as *C. konishii*, *K. davidiana* var. *formosana*, and *T. cryptomerioides*. Moreover, strong migration may also inflict the break-up of locally adapted gene complex. Nonetheless, increased divergence associated with environmental variables at the edge populations of plant species shifting their range altitudinally may provide insights into the adaptive evolution of species. A higher portion of genetic loci was found to be environmentally dependent in *C. formosana* compared with the three other Taiwanese conifers. Additionally, our results revealed the associations of high frequency outlier loci with environmental variables, including altitude-related annual mean temperature and annual temperature range and generally non-altitude-related relative humidity and sunshine hours at elevational margin populations. Relative humidity and sunshine hours generally not altitude specific could have been the major ecological drivers invoked adaptive divergence in *C. formosana*. Results of this study and the previous studies provide insights into the interplay of gene flow and environments in shaping population genetic divergence in Taiwanese conifers.

## Data Availability Statement

The AFLP data used in this study is included in the article/ Supplementary Data Sheet 1.

## Author Contributions

S-YH proposed, funded, and designed the research. W-MC, Y-CC, and S-YH collected the samples. W-MC, C-TC, and S-YH performed the research. S-YH and W-MC wrote the manuscript. All authors analyzed the data and read and approved the final manuscript.

## Disclaimer

Frontiers Media SA remains neutral with regard to jurisdictional claims in published maps and institutional affiliations.

## Conflict of Interest

The authors declare that the research was conducted in the absence of any commercial or financial relationships that could be construed as a potential conflict of interest.

## Footnotes

1 https://asterweb.jpl.nasa.gov/gdem.asp

2 http://lpdaac.usgs.gov

3 https://dbahr.pccu.edu.tw/

## Supplementary Material

Supplementary Figure 1 Minimum cross-entropy and Bayesian information criterion for evaluation of clustering scenarios analyzed using LEA and DAPC.

Supplementary Figure 2 The Spearman’s rank correlation between the 14 retained environmental variables. Aspect (0–360°) and slope (0–90°). BIO1, annual mean temperature; BIO7, annual temperature range; BIO12, annual precipitation; BIO18, precipitation of the warmest quarter; fPAR, fraction of absorbed photosynthetically active radiation; NDVI, normalized difference vegetation index, RainD, number of rainfall days per year; RH, relative humidity; SunH, time of sun shine hours; WSmean, mean wind speed.

Supplementary Table 1 Primer combinations, number of markers, and error rate per locus in AFLP technique.

Supplementary Table 2 The 14 retained site environmental variables of the 12 populations of *Calocedrus formosana.* See Table 1 for abbreviations of the 12 populations of *C. formosana*.

Supplementary Table 3 Summary of Tukey’s *post hoc* pairwise population comparisons of the mean unbiased expected heterozygosity using a linear mixed effect model. In linear mixed effect model, population was treated as a fixed effect and locus as a random effect.

Supplementary Table 4 Pairwise *F*_ST_ between populations of *Calocedrus formosana* using ARLEQUIN with 10,000 permutations.

Supplementary Table 5 *Z* and *q*-values for candidate loci significantly associated with environmental variables identified by the latent factor mixed model (LFMM) approach.

Supplementary Table 6 *P-*values of pairwise population comparisons of the 14 environmental variables using PERMANOVA.

Supplementary Data Sheet 1 Comma-separated value file for scored 437 AFLP markers of the 12 populations (*n* = 243) of *Calocedrus formosana*. The first three columns recorded the coding information of sample ID, population, and forest vegetation zones. The rest of columns represent presence/absence of markers.
