# Supplementary figures and images for: Ecological Factors Generally Not Altitude Related Played Main Roles in Driving Potential Adaptive Evolution at Elevational Range Margin Populations of Taiwan Incense Cedar (Calocedrus formosana)
Source: Front Genet. 2020 Nov 11;11:580630. doi: 10.3389/fgene.2020.580630 (PMC7686793; doi:10.3389/fgene.2020.580630)

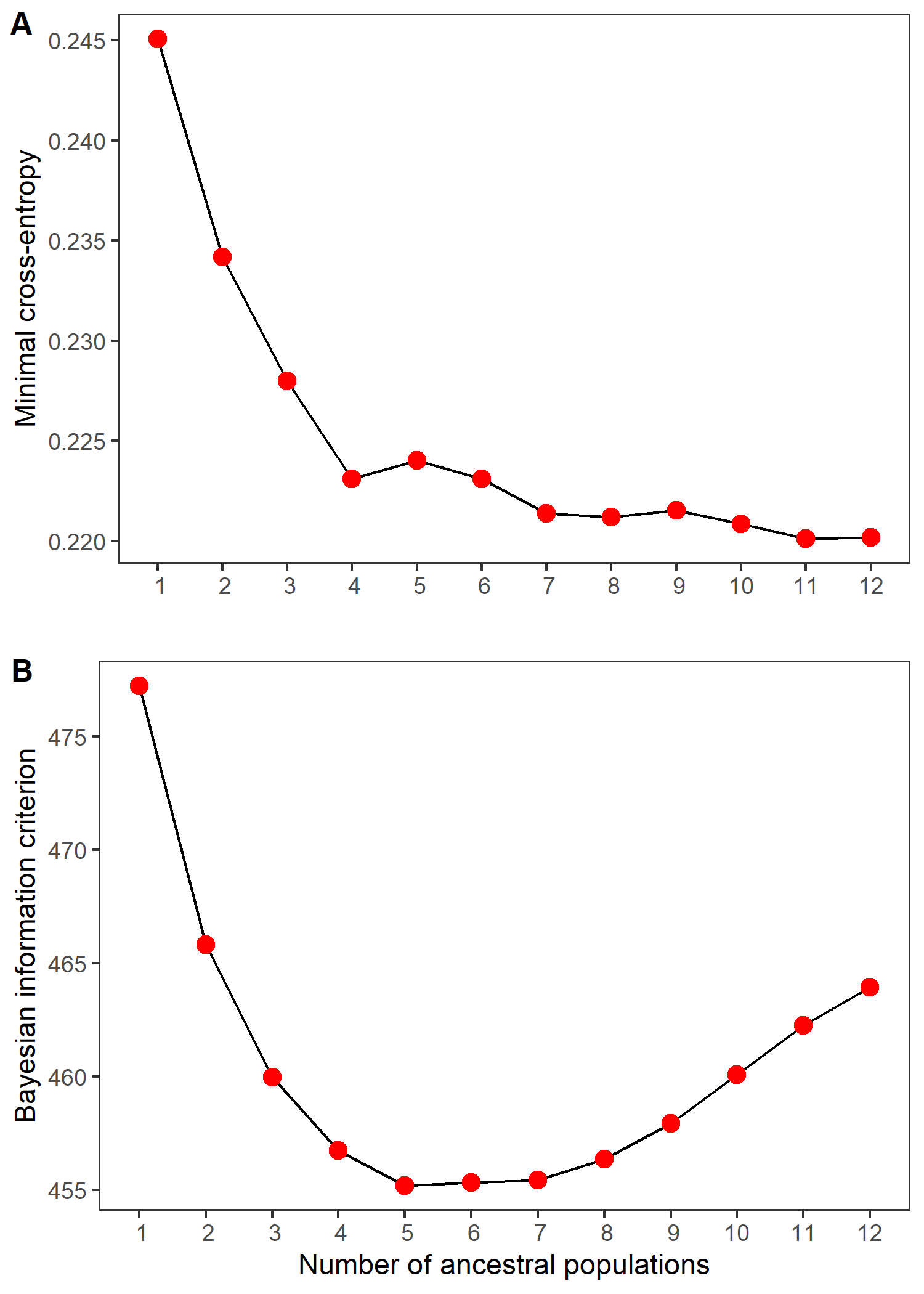

Supplement: Supplementary Figure 1 — Minimum cross-entropy and Bayesian information criterion for evaluation of clustering scenarios analyzed using LEA and DAPC. [file Image_1.TIF]

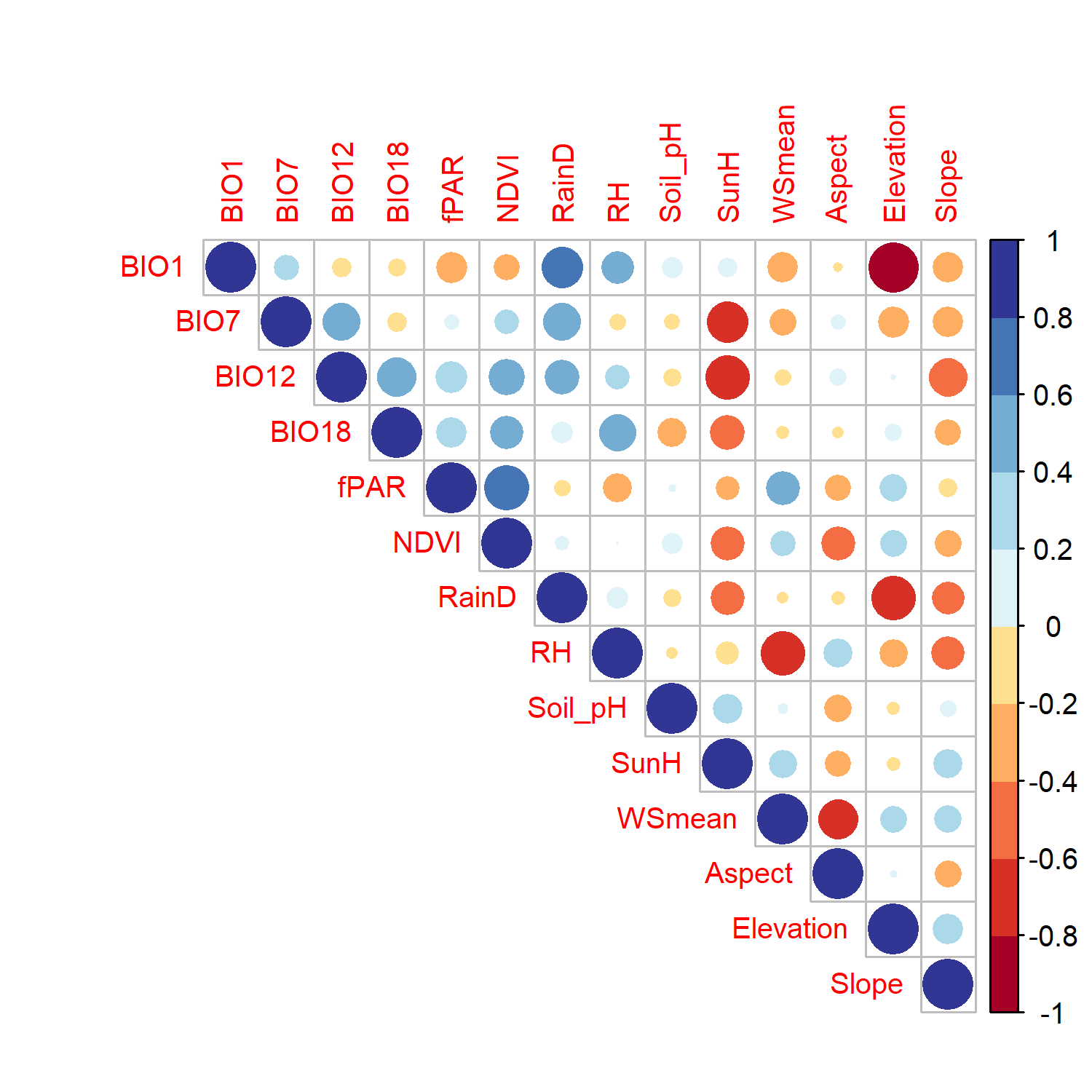

Supplement: Supplementary Figure 2 — The Spearman’s rank correlation between the 14 retained environmental variables. Aspect (0–360°) and slope (0–90°). BIO1, annual mean temperature; BIO7, annual temperature range; BIO12, annual precipitation; BIO18, precipitation of the warmest quarter; fPAR, fraction of absorbed photosynthetically active radiation; NDVI, normalized difference vegetation index, RainD, number of rainfall days per year; RH, relative humidity; SunH, time of sun shine hours; WSmean, mean wind speed. [file Image_2.TIF]
