# Supplementary material for: Ecological Factors Generally Not Altitude Related Played Main Roles in Driving Potential Adaptive Evolution at Elevational Range Margin Populations of Taiwan Incense Cedar (Calocedrus formosana)
Source: Front Genet. 2020 Nov 11;11:580630. doi: 10.3389/fgene.2020.580630 (PMC7686793; doi:10.3389/fgene.2020.580630)
Supplement: Supplementary Table 1 — Primer combinations, number of markers, and error rate per locus in AFLP technique. [file Table_1.DOCX]

**Supplementary Table 1.** Primer combinations, number of markers, and error rate per locus in AFLP technique.

| Primer number and combination | Number of markers | Error rate (%) |
| --- | --- | --- |
|  |  |  |
| 1 E00AAC+M00ACT | 41 | 4.98 |
| 2 E00TAA+M00CTCCA | 33 | 4.82 |
| 3 E00TAA+M00CTGAA | 41 | 4.86 |
| 4 E00TAA+M00GAT | 33 | 4.98 |
| 5 E00TAA+M00GCA | 20 | 4.86 |
| 6 E00TAA+M00GGT | 35 | 4.85 |
| 7 E00TCA+M00CTCTC | 53 | 4.87 |
| 8 E00TCA+M00CTGAA | 53 | 4.99 |
| 9 E00TCA+M00GAT | 34 | 4.93 |
| 10 E00TCA+M00GCA | 32 | 4.88 |
| 11 E00TCA+M00GCG | 62 | 4.98 |
| Total | 437 |  |
| Average | 39.7 (12.0) | 4.91 |

E00 (5’-GACTGCGTACCAATTC-3’)

M00 (5’-GATGAGTCCTGAGTAA-3’)
