# Supplementary material for: Ecological Factors Generally Not Altitude Related Played Main Roles in Driving Potential Adaptive Evolution at Elevational Range Margin Populations of Taiwan Incense Cedar (Calocedrus formosana)
Source: Front Genet. 2020 Nov 11;11:580630. doi: 10.3389/fgene.2020.580630 (PMC7686793; doi:10.3389/fgene.2020.580630)
Supplement: Supplementary Table 2 — The 14 retained site environmental variables of the 12 populations of Calocedrus formosana. See Table 1 for abbreviations of the 12 populations of C. formosana. [file Table_2.DOCX]

**Supplementary Table 2.** The 14 retained site environmental variables of the 12 populations of *Calocedrus formosana.* See **Table 1** for abbreviations of the 12 populations of *Ca. formosana*.

|  | Bioclimate | | | |  | Topology | | |  | Ecology | | | | | | |
| --- | --- | --- | --- | --- | --- | --- | --- | --- | --- | --- | --- | --- | --- | --- | --- | --- |
| Population | BIO1 | BIO7 | BIO12 | BIO18 |  | Aspect | Elevation | Slope |  | fPAR | NDVI | RainD | Soil pH | RH | SunH | WSmean |
| BSS | 149 | 166 | 2484 | 986 |  | 200.3 | 1352 | 31.5 |  | 0.89 | 0.8 | 10.7 | 4.4 | 78.2 | 157.5 | 2.9 |
| CL | 157 | 172 | 2650 | 1091 |  | 175.9 | 1136 | 44.2 |  | 0.9 | 0.85 | 13.4 | 3.5 | 78.7 | 117.3 | 2.4 |
| FCH | 152 | 160 | 3040 | 1515 |  | 239.5 | 1518 | 29.5 |  | 0.87 | 0.84 | 11.5 | 4.5 | 82.4 | 131.1 | 2 |
| HS | 169 | 163 | 2394 | 867 |  | 77.7 | 1243 | 41.4 |  | 0.8 | 0.83 | 11.3 | 6.5 | 78.8 | 158.7 | 2.7 |
| KW | 117 | 168 | 2757 | 1044 |  | 108.4 | 2155 | 30.7 |  | 0.91 | 0.86 | 9.9 | 4.6 | 78.1 | 133 | 2.5 |
| SKL | 111 | 168 | 2792 | 1022 |  | 334.2 | 2209 | 32.9 |  | 0.91 | 0.85 | 10.5 | 4.8 | 78.2 | 125.4 | 2.5 |
| SLS | 169 | 157 | 2128 | 1024 |  | 78.9 | 1220 | 35.5 |  | 0.89 | 0.8 | 12.2 | 4.6 | 76 | 157.3 | 4.8 |
| SML | 196 | 167 | 2230 | 892 |  | 317.6 | 859 | 30.3 |  | 0.4 | 0.71 | 12 | 4.5 | 81.2 | 143.3 | 1.2 |
| SS | 202 | 186 | 2959 | 1140 |  | 51.3 | 436 | 36.1 |  | 0.91 | 0.87 | 13.4 | 5.1 | 78.1 | 113.8 | 2.4 |
| TC | 141 | 165 | 2467 | 979 |  | 335.7 | 1498 | 42.8 |  | 0.75 | 0.72 | 10.8 | 4.6 | 77.9 | 143.9 | 2.3 |
| WL | 185 | 179 | 3655 | 1103 |  | 312.8 | 562 | 13 |  | 0.86 | 0.82 | 14.7 | 4.7 | 79.2 | 116.3 | 2.3 |
| ZL | 191 | 167 | 2427 | 999 |  | 337.4 | 868 | 26 |  | 0.9 | 0.81 | 11.4 | 4.9 | 80.3 | 142.4 | 1.5 |

*Aspect (0–360°) and slope (0–90°). BIO1, annual mean temperature; BIO7, annual temperature range; BIO12, annual precipitation; BIO18, precipitation of the warmest quarter; fPAR, fraction of absorbed photosynthetically active radiation; NDVI, normalized difference vegetation index, RainD, number of rainfall days per year; RH, relative humidity; SunH, time of sun shine hours; WSmean, mean wind speed.*
