# Supplementary material for: Ecological Factors Generally Not Altitude Related Played Main Roles in Driving Potential Adaptive Evolution at Elevational Range Margin Populations of Taiwan Incense Cedar (Calocedrus formosana)
Source: Front Genet. 2020 Nov 11;11:580630. doi: 10.3389/fgene.2020.580630 (PMC7686793; doi:10.3389/fgene.2020.580630)
Supplement: Supplementary Table 3 — Summary of Tukey’s post hoc pairwise population comparisons of the mean unbiased expected heterozygosity using a linear mixed effect model. In linear mixed effect model, population was treated as a fixed effect and locus as a random effect. [file Table_3.DOCX]

**Supplementary Table 3.** Summary of Tukey’s post-hoc pairwise population comparisons of the mean unbiased expected heterozygosity using a linear mixed effect model. In linear mixed effect model, population was treated as a fixed effect and locus as a random effect.

| Population | Pair of vegetation zone | Difference | *t* | *P* |
| --- | --- | --- | --- | --- |
| **BSS- CL** | **MEC-MEC** | -0.03229 | -4.398 | 0.0007 |
| **BSS- FCH** | **MEC-MMC** | 0.030254 | 4.12 | 0.0023 |
| BSS- HS | MEC-MEC | 0.006906 | 0.941 | 0.9987 |
| **BSS- KW** | **MEC-MMC** | -0.04296 | -5.851 | < 0.0001 |
| BSS- SKL | MEC-MMC | -0.01828 | -2.489 | 0.3471 |
| BSS- SLS | MEC-MEC | -0.00887 | -1.208 | 0.9884 |
| BSS- SML | MEC-MEC | 0.01263 | 1.72 | 0.8594 |
| BSS- SS | MEC-SME | 0.007532 | 1.026 | 0.9971 |
| BSS- TC | MEC-MMC | -0.01584 | -2.157 | 0.5816 |
| BSS- WL | MEC-SME | 0.001255 | 0.171 | 1 |
| BSS- ZL | MEC-MEC | -0.00093 | -0.126 | 1 |
| **CL - FCH** | **MEC-MMC** | 0.062548 | 8.518 | < 0.0001 |
| **CL - HS** | **MEC-MEC** | 0.0392 | 5.338 | < 0.0001 |
| CL - KW | MEC-MMC | -0.01067 | -1.453 | 0.9527 |
| CL - SKL | MEC-MMC | 0.014017 | 1.909 | 0.7539 |
| CL - SLS | MEC-MEC | 0.02342 | 3.189 | 0.0637 |
| **CL - SML** | **MEC-MEC** | 0.044923 | 6.118 | < 0.0001 |
| **CL - SS** | **MEC-SME** | 0.039825 | 5.423 | < 0.0001 |
| CL - TC | MEC-MMC | 0.016458 | 2.241 | 0.5194 |
| **CL - WL** | **MEC-SME** | 0.033548 | 4.569 | 0.0003 |
| CL - ZL | MEC-MEC | 0.031367 | 4.272 | 0.0012 |
| FCH - HS | MMC-MEC | -0.02335 | -3.18 | 0.0656 |
| **FCH - KW** | **MMC-MMC** | -0.07322 | -9.971 | < 0.0001 |
| **FCH - SKL** | **MMC-MMC** | -0.048530 | -6.609 | < 0.0001 |
| **FCH - SLS** | **MMC-MEC** | -0.039128 | -5.328 | < 0.0001 |
| FCH - SML | MMC-MEC | -0.017625 | -2.4 | 0.406 |
| FCH - SS | MMC-SME | -0.02272 | -3.094 | 0.0838 |
| **FCH - TC** | **MMC-MMC** | -0.04609 | -6.277 | < 0.0001 |
| **FCH - WL** | **MMC-SME** | -0.029 | -3.949 | 0.0045 |
| **FCH - ZL** | **MMC-MEC** | -0.03118 | -4.246 | 0.0013 |
| **HS - KW** | **MEC-MMC** | -0.04987 | -6.791 | < 0.0001 |
| **HS - SKL** | **MEC-MMC** | -0.02518 | -3.429 | 0.0301 |
| HS - SLS | MEC-MEC | -0.01578 | -2.149 | 0.5872 |
| HS - SML | MEC-MEC | 0.005723 | 0.779 | 0.9998 |
| HS - SS | MEC-SME | 0.000626 | 0.085 | 1 |
| HS - TC | MEC-MMC | -0.02274 | -3.097 | 0.0832 |
| HS - WL | MEC-SME | -0.00565 | -0.77 | 0.9998 |
| HS - ZL | MEC-MEC | -0.00783 | -1.067 | 0.996 |
| **KW - SKL** | **MMC-MMC** | 0.024688 | 3.362 | 0.0374 |
| **KW - SLS** | **MMC-MEC** | 0.03409 | 4.642 | 0.0002 |
| **KW - SML** | **MMC-MEC** | 0.055593 | 7.571 | < 0.0001 |
| **KW - SS** | **MMC-SME** | 0.050496 | 6.877 | < 0.0001 |
| **KW - TC** | **MMC-MMC** | 0.027128 | 3.694 | 0.012 |
| **KW - WL** | **MMC-SME** | 0.044219 | 6.022 | < 0.0001 |
| **KW - ZL** | **MMC-MEC** | 0.042037 | 5.725 | < 0.0001 |
| SKL - SLS | MMC-MEC | 0.009403 | 1.28 | 0.9816 |
| **SKL - SML** | **MMC-MEC** | 0.030906 | 4.209 | 0.0016 |
| **SKL - SS** | **MMC-SME** | 0.025808 | 3.515 | 0.0226 |
| SKL - TC | MMC-MMC | 0.00244 | 0.332 | 1 |
| SKL - WL | MMC-SME | 0.019531 | 2.66 | 0.247 |
| SKL - ZL | MMC-MEC | 0.017349 | 2.363 | 0.432 |
| SLS - SML | MEC-MEC | 0.021503 | 2.928 | 0.1312 |
| SLS - SS | MEC-SME | 0.016405 | 2.234 | 0.5246 |
| SLS - TC | MEC-MMC | -0.00696 | -0.948 | 0.9986 |
| SLS - WL | MEC-SME | 0.010128 | 1.379 | 0.9675 |
| SLS - ZL | MEC-MEC | 0.007947 | 1.082 | 0.9954 |
| SML - SS | MEC-SME | -0.0051 | -0.694 | 0.9999 |
| **SML - TC** | **MEC-MMC** | -0.02847 | -3.876 | 0.006 |
| SML - WL | MEC-SME | -0.01138 | -1.549 | 0.9267 |
| SML - ZL | MEC-MEC | -0.01356 | -1.846 | 0.7922 |
| SS - TC | SE-MMC | -0.02337 | -3.182 | 0.0651 |
| SS - WL | SE-SME | -0.00628 | -0.855 | 0.9995 |
| SS - ZL | SE-MEC | -0.00846 | -1.152 | 0.9922 |
| TC - WL | MMC-SME | 0.01709 | 2.327 | 0.457 |
| TC - ZL | MMC-MEC | 0.014909 | 2.03 | 0.6726 |
| WL - ZL | SME-MEC | -0.00218 | -0.297 | 1 |

*See Table 1 for population codes.*

*Populations SS and WL located in sub-montane evergreen zone (SME, 0-800 m); populations BSS, CL, HS, SLS, SML, and ZL located in montane evergreen cloud zone (MEC, 800-1400 m); and populations FCH, KW, SKL, and TC located in montane mixed cloud zone (MMC, 1400m~).*

*Bold letters indicate significant pair comparisons between populations and between vegetation zones.*
