# Supplementary material for: Ecological Factors Generally Not Altitude Related Played Main Roles in Driving Potential Adaptive Evolution at Elevational Range Margin Populations of Taiwan Incense Cedar (Calocedrus formosana)
Source: Front Genet. 2020 Nov 11;11:580630. doi: 10.3389/fgene.2020.580630 (PMC7686793; doi:10.3389/fgene.2020.580630)
Supplement: Supplementary Table 4 — Pairwise FST between populations of Calocedrus formosana using ARLEQUIN with 10,000 permutations. [file Table_4.DOCX]

**Supplementary Table 4.** Pairwise *F*_ST_ (below diagonal) and *P* values (above diagonal) between populations of *Calocedrus formosana* based on the total data using ARLEQUIN with 10,000 permutations.

|  | BSS | CL | FCH | HS | KW | SKL | SLS | SML | SS | TC | WL | ZL |
| --- | --- | --- | --- | --- | --- | --- | --- | --- | --- | --- | --- | --- |
| BSS |  | 0 | 0 | 0.00257 | 0 | 0 | 0 | 0 | 0 | 0 | 0 | 0 |
| CL | 0.17251 |  | 0 | 0 | 0.34937 | 0.0096 | 0.0002 | 0 | 0.00762 | 0 | 0.0002 | 0 |
| FCH | 0.18121 | 0.26235 |  | 0 | 0 | 0 | 0 | 0.0003 | 0 | 0 | 0 | 0 |
| HS | 0.02502 | 0.17878 | 0.18479 |  | 0 | 0 | 0 | 0 | 0 | 0.0001 | 0 | 0 |
| KW | 0.20647 | 0.00318 | 0.27072 | 0.2024 |  | 0 | 0 | 0 | 0.0003 | 0 | 0 | 0 |
| SKL | 0.15247 | 0.04007 | 0.23614 | 0.16877 | 0.07033 |  | 0 | 0 | 0.02594 | 0 | 0.0004 | 0 |
| SLS | 0.19622 | 0.10203 | 0.27647 | 0.19197 | 0.13505 | 0.07723 |  | 0 | 0.03267 | 0 | 0.0003 | 0 |
| SML | 0.14301 | 0.18492 | 0.03734 | 0.14662 | 0.19447 | 0.17447 | 0.2148 |  | 0 | 0 | 0 | 0 |
| SS | 0.13524 | 0.09012 | 0.26441 | 0.14918 | 0.11851 | 0.03443 | 0.03552 | 0.19386 |  | 0 | 0.4949 | 0.0001 |
| TC | 0.03973 | 0.13914 | 0.16063 | 0.0382 | 0.1682 | 0.12837 | 0.12716 | 0.11381 | 0.09579 |  | 0 | 0 |
| WL | 0.13897 | 0.074 | 0.22896 | 0.1575 | 0.11965 | 0.03983 | 0.06372 | 0.16169 | -0.00229 | 0.1049 |  | 0 |
| ZL | 0.1496 | 0.17349 | 0.11922 | 0.14447 | 0.19721 | 0.16594 | 0.19739 | 0.05289 | 0.16141 | 0.11605 | 0.14545 |  |
