# Supplementary material for: Ecological Factors Generally Not Altitude Related Played Main Roles in Driving Potential Adaptive Evolution at Elevational Range Margin Populations of Taiwan Incense Cedar (Calocedrus formosana)
Source: Front Genet. 2020 Nov 11;11:580630. doi: 10.3389/fgene.2020.580630 (PMC7686793; doi:10.3389/fgene.2020.580630)
Supplement: Supplementary Table 5 — Z and q-values for candidate loci significantly associated with environmental variables identified by the latent factor mixed model (LFMM) approach. [file Table_5.DOCX]

**Supplementary Table 5.** *Z* and *q* values for candidate loci significantly associated with environmental variables identified by the latent factor mixed model (LFMM) approach.

| Locus | *Z* (*q*) value | | | | | | | | | | | | | | | |
| --- | --- | --- | --- | --- | --- | --- | --- | --- | --- | --- | --- | --- | --- | --- | --- | --- |
|  | BIO1 | BIO7 | BIO12 | BIO18 |  | Aspect | Elevation | Slope |  | fPAR | NDVI | RainD | RH | Soil pH | SunH | WSmean |
| aP11_1650 | 1.732 (0.003866) | 0.786 (0.000352) | 1.101  (9.06E-06) | 1.638  (1.34E-07) |  | 0.546 (0.186518) | 1.654  (3.18E-05) | 0.598 (0.580395) |  | 1.748  (2.77E-08) | 0.444 (0.611142) | 0.629 (0.015634) | 5.671 (9.45E-54) | 0.632 (0.020668) | 1.62 (0.000454) | 1.166 (0.000238) |
| aP11_1984 | 0.932 (0.161343) | 0.247 (0.354206) | 0.049 (0.718171) | 2.161  (8.34E-13) |  | 0.407 (0.392455) | 1.031 (0.014686) | 0.559 (0.64587) |  | 1.539  (1.14E-06) | 0.781 (0.415003) | 0.092 (0.637831) | 3.169 (3.27E-17) | 0.765 (0.003561) | 2.057 (4.81E-06) | 0.251 (0.5137) |
| aP11_2003 | 0.279 (0.509492) | 0.366 (0.13767) | 0.943 (0.000199) | 1.316  (3.75E-05) |  | 0.584 (0.145548) | 0.958 (0.024223) | 0.105 (0.987592) |  | 1.399  (1.11E-05) | 0.014 (0.888066) | 0.247 (0.401304) | 3.192 (2.06E-17) | 0.253 (0.553570) | 0.978 (0.042050) | 0.484 (0.188422) |
| aP11_2338 | 0.285 (0.509492) | 0.364 (0.139585) | 0.527 (0.063444) | 1.231 (0.000141) |  | 0.065 (0.989082) | 0.861 (0.046889) | 0.187 (0.941505) |  | 1.504  (1.90E-06) | 0.062 (0.874628) | 0.061 (0.65575) | 3.864 (4.13E-25) | 0.244 (0.583515) | 1.316 (0.004636) | 0.768 (0.024119) |
| aP11_4330 | 0.828 (0.222679) | 0.447 (0.057785) | 0.545 (0.057139) | 1.196 (0.000235) |  | 0.468 (0.293921) | 1.619  (4.72E-05) | 0.29 (0.841984) |  | 1.747  (2.77E-08) | 0.225 (0.816392) | 0.108 (0.62632) | 8.039 (8.05E-108) | 0.28 (0.468512) | 0.135 (0.690473) | 1.324 (2.36E-05) |
| aP24_1967 | 1.697 (0.004750) | 0.716 (0.001202) | 0.107 (0.646119) | 0.032 (0.955504) |  | 0.488 (0.261053) | 2.286  (3.47E-09) | 0.31 (0.818059) |  | 0.258 (0.393490) | 1.772 (0.005824) | 0.482 (0.076395) | 0.922 (0.022926) | 0.209 (0.667384) | 1.532 (0.000934) | 0.78 (0.0220044) |
| aP24_3746 | 2.323  (6.05E-05) | 0.318 (0.215870) | 0.623 (0.023331) | 2.071  (8.66E-12) |  | 0.866 (0.014782) | 1.183 (0.004540) | 1.86  (0.00123) |  | 1.942  (4.87E-10) | 0.317 (0.717886) | 0.045 (0.667516) | 1.602 (4.58E-05) | 1.071 (2.03E-05) | 1.222 (0.009427) | 1.15 (0.000294) |
| aP34_1606 | 1.117 (0.084579) | 1.834  (1.02E-18) | 0.746 (0.004845) | 0.927 (0.006216) |  | 0.89 (0.012616) | 0.03 (0.7518929) | 0.60 (0.5803954) |  | 2.012  (1.22E-10) | 0.611 (0.564260) | 0.58 (0.028804) | 2.191 (1.08E-08) | 0.496 (0.087427) | 1.532 (0.000934) | 0.163 (0.612461) |
| aP34_1681 | 2.189 (0.000169) | 0.701 (0.001497) | 0.443 (0.139274) | 0.356 (0.504337) |  | 0.617 (0.115672) | 1.433 (0.00040) | 0.935 (0.203310) |  | 0.616 (0.065810) | 0.004 (0.89081) | 0.507 (0.059038 | 0.232 (0.362172) | 0.351 (0.301227) | 1.529 (0.000935) | 0.315 (0.445299) |
| aP34_1762 | 3.2  (4.20E-08) | 1.557  (1.00E-13) | 0.965 (0.000143) | 0.382 (0.451653) |  | 0.447 (0.325290) | 2.664  (2.46E-12) | 2.936  (1.29E-08) |  | 1.091 (0.000781) | 0.554 (0.574292) | 0.614 (0.01876) | 3.851 (4.85E-25) | 0.084 (0.956870) | 0.238 (0.624311) | 4.308 (7.24E-50) |
| aP34_2113 | 2.38  (4.00E-05) | 1.14  (7.80E-08) | 0.58 (0.039525) | 1.131 (0.000585) |  | 0.083 (0.966180) | 0.863 (0.046506) | 0.922 (0.213119) |  | 1.605  (3.78E-07) | 0.811 (0.381119) | 0.186 (0.514488) | 1.794 (3.74E-06) | 0.83 (0.001450) | 1.133 (0.016510) | 0.137 (0.650255) |
| aP34_2507 | 1.351 (0.028583) | 2.188  (4.60E-26) | 0.781 (0.002882) | 0.026 (0.958337) |  | 0.868 (0.014782) | 0.452 (0.347277) | 0.275 (0.848818) |  | 0.366 (0.256752) | 1.439 (0.029253) | 0.998  (7.15E-05) | 1.626 (3.53E-05) | 0.761 (0.003748) | 2.27 (3.70E-07) | 0.973 (0.002854) |
| aP34_2811 | 0.737 (0.275302) | 1.74  (5.87E-17) | 0.861 (0.000875) | 0.199 (0.72327) |  | 0.545 (0.186518) | 0.227 (0.564402) | 0.854 (0.273291) |  | 0.957 (0.003311) | 1.609 (0.011032) | 0.703 (0.006561) | 1.328 (0.000845) | 0.96 (0.000170) | 1.978 (1.11E-05) | 0.851 (0.010595) |
| aP34_3008 | 2.465  (2.15E-05) | 0.065 (0.633068) | 0.053 (0.717035) | 0.31 (0.5778821) |  | 1.325  (6.14E-05) | 1.75  (8.60E-06) | 0.997 (0.163267) |  | 1.217 (0.000149) | 0.926 (0.264169) | 0.895 (0.000410) | 0.363 (0.288280) | 0.154 (0.83674) | 0.28 (0.605180) | 0.598 (0.089335) |
| aP35_2216 | 0.278 (0.509492) | 1.716  (1.56E-16) | 0.008 (0.746267) | 0.781 (0.027944) |  | 0.961 (0.005974) | 1.267 (0.001968) | 0.485 (0.668910) |  | 1.006 (0.002018) | 0.077 (0.872296) | 0.762 (0.003183) | 0.571 (0.150427) | 1.759 (1.60E-13) | 4.648 (1.05E-28) | 0.258 (0.513676) |
| aP38_2271 | 1.451 (0.019115) | 0.229 (0.386712) | 0.537 (0.06056) | 0.741 (0.040050) |  | 0.219 (0.751932) | 1.662  (2.96E-05) | 0.523 (0.668910) |  | 1.514  (1.64E-06) | 0.374 (0.678567) | 0.234 (0.429322) | 4.011 (6.26E-27) | 0.201 (0.686457) | 0.022 (0.71756) | 1.245 (7.73E-05) |
| aP47_3520 | 2.305  (6.36E-05) | 0.599 (0.007399) | 0.058 (0.712880) | 0.315 (0.57747) |  | 0.668 (0.078574) | 1.795  (5.63E-06) | 1.552 (0.010725) |  | 0.548 (0.105005) | 0.338 (0.703892) | 0.566 (0.033120) | 0.448 (0.223881) | 0.771 (0.003306) | 1.328 (0.004219) | 0.47 (0.2044779) |
| aP49_1747 | 1.724 (0.003993) | 0.08 (0.607186) | 0.208 (0.500148) | 1.454  (3.94E-06) |  | 0.03 (0.992268) | 1.337 (0.000991) | 0.886 (0.251672) |  | 2.899  (3.14E-21) | 0.531 (0.574292) | 0.477 (0.077250) | 1.304 (0.001027) | 1.213 (9.33E-07) | 0.471 (0.401085) | 1.329 (2.23E-05) |
| aP49_2083 | 1.267 (0.043361) | 1.226  (7.15E-09) | 0.721 (0.006866) | 0.215 (0.685767) |  | 0.108 (0.926389) | 0.241 (0.552454) | 0.287 (0.843442) |  | 1.149 (0.000364) | 1.643 (0.010358) | 0.653 (0.01215) | 0.302 (0.328616) | 0.536 (0.058392) | 2.21 (8.05E-07) | 0.702 (0.040824) |
| aP49_2597 | 2.181 (0.000174) | 0.37 (0.133290) | 0.211 (0.500148) | 0.49 (0.244385) |  | 1.296  (9.22E-05) | 1.417 (0.000448) | 1.509 (0.012596) |  | 0.71 (0.032756) | 0.017 (0.886256) | 0.759 (0.003260) | 0.336 (0.307469) | 0.479 (0.104056) | 1.134 (0.016510) | 0.513 (0.156501) |
| aP49_2698 | 0.753 (0.265434) | 1.148  (6.30E-08) | 0.359 (0.242542) | 0.774 (0.028891) |  | 0.759 (0.038161) | 0.592 (0.211180) | 0.126 (0.987592) |  | 1.573  (6.66E-07) | 0.948 (0.249257) | 0.696 (0.007099) | 2.193 (1.08E-08) | 0.323 (0.364133) | 0.774 (0.123205) | 1.098 (0.000570) |
| aP55_1883 | 0.813 (0.226946) | 1.385  (4.65E-11) | 0.386 (0.216286) | 0.357 (0.504337) |  | 0.533 (0.198038) | 0.734 (0.098363) | 0.085 (0.987592) |  | 0.343 (0.282370) | 1.416 (0.030941) | 0.651 (0.012365) | 0.436 (0.228922) | 0.532 (0.060027) | 1.416 (0.002208) | 1.585 (3.56E-07) |
| aP56_1840 | 0.022 (0.602274) | 0.234 (0.383138) | 0.082 (0.685072) | 1.865  (1.19E-09) |  | 0.705 (0.058938) | 0.505 (0.292219) | 0.47 (0.668910) |  | 0.412 (0.212864) | 1.696 (0.008292) | 0.101 (0.630027) | 2.593 (1.04E-11) | 1.218 (8.79E-07) | 3.738 (1.48E-18) | 0.47 (0.204478) |
| aP57_1778 | 0.319 (0.488340) | 1.31  (5.92E-10) | 0.082 (0.685072) | 0.266 (0.613678) |  | 0.104 (0.926389) | 0.038 (0.747953) | 0.037 (0.987592) |  | 0.9 (0.0057286) | 1.169 (0.097758) | 0.272 (0.363933) | 0.517 (0.178834) | 0.939 (0.000243) | 1.055 (0.026469) | 1.469 (2.33E-06) |
| aP57_2129 | 2.606  (8.41E-06) | 0.254 (0.34088) | 0.448 (0.134057) | 1.435  (5.26E-06) |  | 0.406 (0.393444) | 1.783  (5.88E-06) | 1.138 (0.093244) |  | 0.882 (0.006799) | 0.002 (0.890808) | 0.975 (0.000109) | 1.197 (0.002705) | 0.073 (0.971818) | 0.336 (0.556385) | 0.373 (0.345907) |
| aP57_3127 | 0.634 (0.357931) | 1.217  (9.21E-09) | 0.269 (0.407655) | 0.351 (0.507236) |  | 0.423 (0.362864) | 0.804 (0.068138) | 1.249 (0.05926) |  | 1.184 (0.000230) | 1.07 (0.1493790) | 0.056 (0.658600) | 1.125 (0.005255) | 1.168 (2.53E-06) | 1.245 (0.008102) | 0.35 (0.380494) |
