# Supplementary material for: Ecological Factors Generally Not Altitude Related Played Main Roles in Driving Potential Adaptive Evolution at Elevational Range Margin Populations of Taiwan Incense Cedar (Calocedrus formosana)
Source: Front Genet. 2020 Nov 11;11:580630. doi: 10.3389/fgene.2020.580630 (PMC7686793; doi:10.3389/fgene.2020.580630)
Supplement: Supplementary Table 6 — P-values of pairwise population comparisons of the 14 environmental variables using PERMANOVA. [file Table_6.DOCX]

**Supplementary Table 6.** *P* values of pairwise population comparisons of the 14 retained environmental variables using PERMANOVA.

|  | BSS | CL | FCH | HS | KW | SKL | SLS | SML | SS | TC | WL |
| --- | --- | --- | --- | --- | --- | --- | --- | --- | --- | --- | --- |
| CL | 0.0021 |  |  |  |  |  |  |  |  |  |  |
| FCH | 0.0021 | 0.0021 |  |  |  |  |  |  |  |  |  |
| HS | 1 | 0.0021 | 0.0021 |  |  |  |  |  |  |  |  |
| KW | 0.0021 | 0.0021 | 0.0021 | 1 |  |  |  |  |  |  |  |
| SKL | 1 | 1 | 0.0021 | 1 | 0.0021 |  |  |  |  |  |  |
| SLS | 0.0021 | 1 | 1 | 1 | 1 | 0.0021 |  |  |  |  |  |
| SML | 1 | 0.0021 | 1 | 0.0021 | 0.0021 | 0.0021 | 1 |  |  |  |  |
| SS | 1 | 1 | 1 | 1 | 0.0021 | 0.0021 | 1 | 1 |  |  |  |
| TC | 1 | 0.0021 | 0.0021 | 1 | 1 | 0.0021 | 0.0021 | 0.0021 | 0.0021 |  |  |
| WL | 1 | 1 | 1 | 1 | 0.0021 | 1 | 0.0021 | 0.0021 | 1 | 1 |  |
| ZL | 0.0021 | 0.0021 | 0.0021 | 1 | 1 | 0.0021 | 0.0021 | 1 | 1 | 1 | 1 |

*Significance determined by 999 permutations and a false discovery rate of 5%.*

*Populations SS and WL located in sub-montane evergreen zone (SME, 0-800 m); populations BSS, CL, HS, SLS, SML, and ZL located in montane evergreen cloud zone (MEC, 800-1400 m); and populations FCH, KW, SKL, and TC located in montane mixed cloud zone (MMC, 1400m~).*
